# Identification of the decumbenone biosynthetic gene cluster in *Penicillium decumbens* and the importance for production of calbistrin

**DOI:** 10.1186/s40694-018-0063-4

**Published:** 2018-12-19

**Authors:** Sietske Grijseels, Carsten Pohl, Jens Christian Nielsen, Zahida Wasil, Yvonne Nygård, Jens Nielsen, Jens C. Frisvad, Kristian Fog Nielsen, Mhairi Workman, Thomas Ostenfeld Larsen, Arnold J. M. Driessen, Rasmus John Normand Frandsen

**Affiliations:** 1 Department of Biotechnology and Biomedicine, Technical University of Denmark, 2800 Kgs. Lyngby, Denmark; 2 Molecular Microbiology, Groningen Biomolecular Sciences and Biotechnology Institute, University of Groningen, 9747 AG Groningen, The Netherlands; 3 Department of Biology and Biological Engineering, Chalmers University of Technology, 412 96 Gothenburg, Sweden; 4 Novo Nordisk Foundation Center for Biosustainability, Technical University of Denmark, 2800 Kgs. Lyngby, Denmark

**Keywords:** *Penicillium decumbens*, Calbistrin, Secondary metabolite, Decalin, Polyketide, Biosynthesis

## Abstract

**Background:**

Filamentous fungi are important producers of secondary metabolites, low molecular weight molecules that often have bioactive properties. Calbistrin A is a secondary metabolite with an interesting structure that was recently found to have bioactivity against leukemia cells. It consists of two polyketides linked by an ester bond: a bicyclic decalin containing polyketide with structural similarities to lovastatin, and a linear 12 carbon dioic acid structure. Calbistrin A is known to be produced by several uniseriate black Aspergilli, *Aspergillus versicolor*-related species, and Penicillia. *Penicillium decumbens* produces calbistrin A and B as well as several putative intermediates of the calbistrin pathway, such as decumbenone A-B and versiol.

**Results:**

A comparative genomics study focused on the polyketide synthase (PKS) sets found in three full genome sequence calbistrin producing fungal species, *P. decumbens, A. aculeatus* and *A. versicolor*, resulted in the identification of a novel, putative 13-membered calbistrin producing gene cluster (*calA* to *calM*). Implementation of the CRISPR/Cas9 technology in *P. decumbens* allowed the targeted deletion of genes encoding a polyketide synthase (*calA*), a major facilitator pump (*calB*) and a binuclear zinc cluster transcription factor (*calC*). Detailed metabolic profiling, using UHPLC-MS, of the ∆*calA* (PKS) and ∆*calC* (TF) strains confirmed the suspected involvement in calbistrin productions as neither strains produced calbistrin nor any of the putative intermediates in the pathway. Similarly analysis of the excreted metabolites in the ∆*calB* (MFC-pump) strain showed that the encoded pump was required for efficient export of calbistrin A and B.

**Conclusion:**

Here we report the discovery of a gene cluster (*calA*-*M*) involved in the biosynthesis of the polyketide calbistrin in *P. decumbens*. Targeted gene deletions proved the involvement of CalA (polyketide synthase) in the biosynthesis of calbistrin, CalB (major facilitator pump) for the export of calbistrin A and B and CalC for the transcriptional regulation of the *cal*-cluster. This study lays the foundation for further characterization of the calbistrin biosynthetic pathway in multiple species and the development of an efficient calbistrin producing cell factory.

**Electronic supplementary material:**

The online version of this article (10.1186/s40694-018-0063-4) contains supplementary material, which is available to authorized users.

## Background

Filamentous fungi are generally prolific producers of secondary metabolites, which possess a wide range of different biological activities. It is a widely accepted view that secondary metabolites serve an important role for the producing fungi to survive in their respective ecological niches, yet many of these small-molecules are also of great importance to humans. Prominent examples of medical use of secondary metabolites include the antibacterial penicillin, the cholesterol-lowering agent lovastatin/compactin and the antifungal griseofulvin. Today fungal secondary metabolites continue to serve as an important source of small-molecules for the discovery of novel drugs.

The amounts of secondary metabolites that are naturally produced by fungi are often far below the amounts necessary for profitable industrial-scale production of the given compound. Traditionally, native fungal production strains have been optimized via strategies relying on random mutagenesis coupled with screening for strains with improved production levels and fermentations properties. The most well-known example being the optimization of penicillin production, where strain improvement programs have succeeded in increasing titers and productivity by at least three orders of magnitude [1]. Recent advances in our understanding of the metabolic pathways for the production of secondary metabolites, full genome sequences, and improvements in genetic engineering tools now allow rational strain improvement by metabolic engineering for enhancing the natural product yield [2–4]. However, in order to employ such techniques, the biosynthetic genes and/or regulatory elements for production of a given compound first have to be identified and characterized. Over the past decades, the genetic basis for production of numerous fungal secondary metabolites has been elucidated, by linking production to gene clusters or genes encoding key-enzyme responsible for biosynthesis of the carbon backbone of the respective secondary metabolites. Still, for the vast majority of the secondary metabolites known today, the biosynthetic pathway and genetic basis remains unknown.

**Fig. 1:**
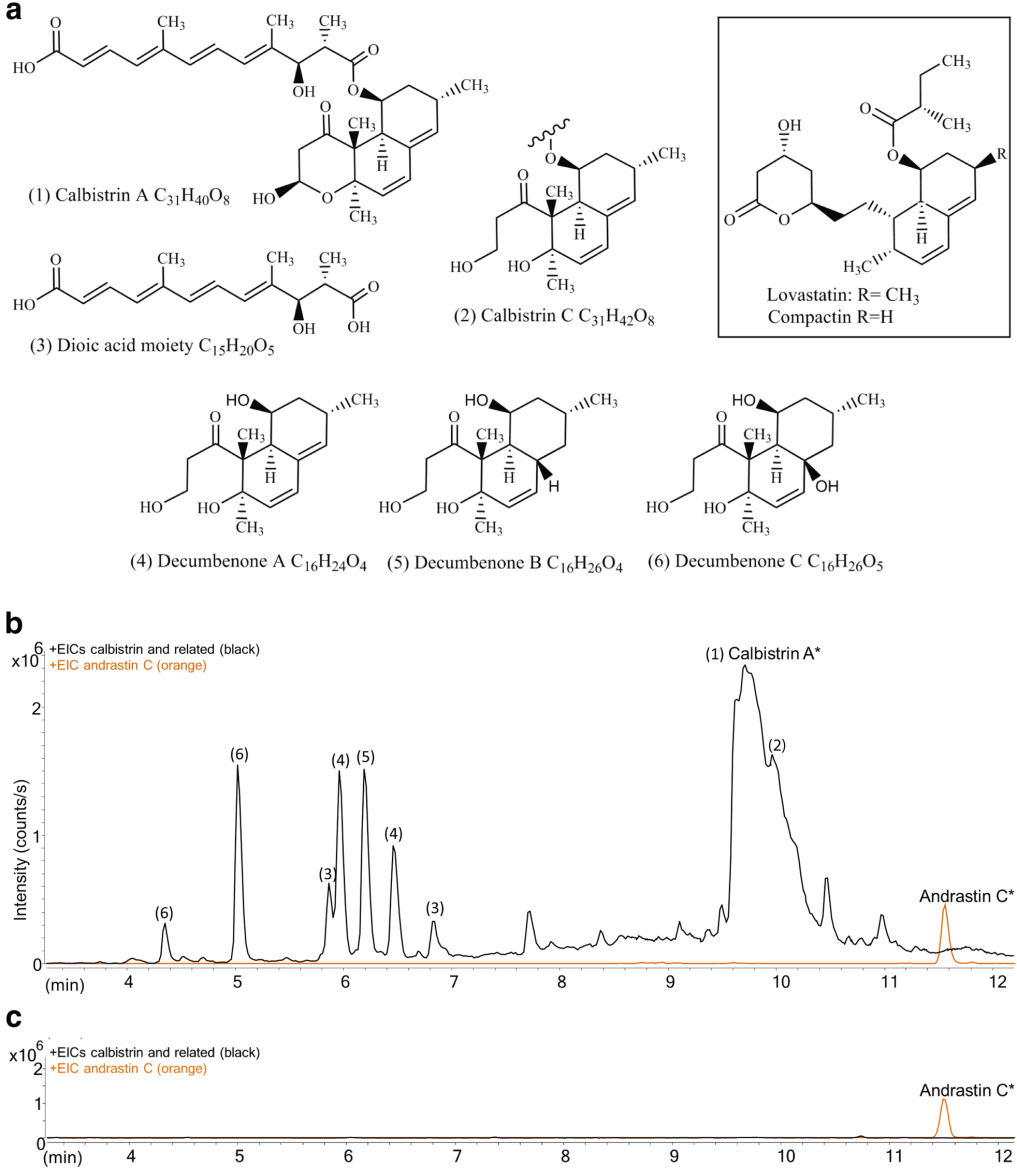
Chemical structures of calbistrin and related metabolites and UHPLC-HRMS analysis of *P. decumbens* wild type and PKS mutant strains. **a** Chemical structures of (1) calbistrin A, (2) calbistrin C, (3) putative linear moiety, (4) decumbenone A, (5) decumbenone B, (6) decumbenone C, and in the box compactin and lovastatin. **b** UHPLC-HRMS analysis of the wild type *P. decumbens* culture extract. Merged extracted ion chromatograms (EICs), ± m/z 0.005 of molecular features detected for compounds 1–6: 263.1642; 281.1742; 321.1670; 337.1401; 245.1177; 303.1204; 247.1697; 265.1806; 305.1720; 245.1538; 303.1575; 319.1331; 505.2591; 523.2705; 563.2622; 525.2847; 565.2776; and 285.1463. Additionally, the EIC of andrastin C (m/z 473.2898) is shown in orange. Calbistrin A and andrastin C were confirmed with a reference standard (marked with *), the other compounds were tentative identified based on UV-spectra and MS/HRMS fragmentation patterns. **c** UHPLC-HRMS results of *P. decumbens* ∆PKS culture extract. Merged EICs of molecular features detected for compounds 1-7 and EIC of andrastin C as in B

The secondary metabolites calbistrin A has been reported to possess a number of interesting bioactivities such as antifungal active against *Candida albicans* [5], 3-hydroxy-3-methyl-glutaryl-coenzyme A reductase inhibition in mammalian cells [6] and cytotoxic toward both healthy and leukemic human cells [7]. Calbistrin A and the related B and C are produced by several uniseriate black Aspergilli, *Aspergillus versicolor*-related species and Penicillia species [8, 9]. Among the Penicillia, the recently genome sequenced *Penicillium decumbens* [10] is interesting because it produces calbistrin A and C and also accumulates several metabolites that are structurally related to calbistrins, namely decumbenone A, B and C [11] (Fig. 1a). All calbistrins are predicted to consist of two individual polyketide chains linked by an ester bond: a decalin containing heptaketide (C14 chain) and a linear dioic acid (also termed dicarboxylic acid) structure formed from a hexaketide (C12 chain) [8]. The calbistrins show structural similarities to the natural cholesterol lowering statins, such as lovastatin produced by *Monascus ruber* [12] and *A. terreus* [13] and compactin produced by *P. solitum* [14–16]. Compactin and lovastatin are both known to consist of two separately synthesized polyketides, a decalin structure formed from a nonaketide (C18 chain) and an ester bound linear diketide (C4 chain) attached to the decalin structure at the same position as seen in calbistrins (Fig. 1). Biosynthesis of the two natural statins is well documents in literature, and formation of the decalin structure has been shown to proceed via an enzymatic intramolecular [4 + 2] Diels–Alder cycloaddition, catalyzed by the polyketide synthase (PKS) responsible formation of the nonaketide backbone of these molecules [17].

Motivated by the reported activities of calbistrin A, the interesting structural similarities and differences between the calbistrins and naturally occuring statins we set out to elucidate the genetic and enzymatic basis for biosynthesis of calbistrins. We chose to perform a comparative genomic analysis of known calbistrin producers, which resulted in the identification of a putative biosynthetic gene cluster (*cal*) for production of calbistrin. To prove the suggested involvement of the identified genes we next developed a transformation protocol and a CRISPR/Cas9 based system for targeted genetic modification of *P. decumbens.* This system allowed us to efficiently delete three genes in the putative *cal*-cluster and analyze the metabolic effects. Deletion of a putative PKS (*calA*) and a transcription factor (*calC*) resulted in the complete abolishment of calbistrin biosynthesis, while deletion of a putative efflux pump (*calB*) significantly reduced extracellular levels of calbistrin A and C. The presented results lay the foundation for the future optimization and development of an efficient cell factory for the production of calbistrins.

## Results

### Chemical analysis reveals the presence of calbistrins and related compounds in extracts of *P. decumbens*

Ultra high performance liquid chromatography-high resolution-mass spectrometry (UHPLC-HRMS) analysis of ethyl acetate extracts of the *P. decumbens* wild-type (WT) cultured on Czapek yeast autolysate medium (CM) showed that calbistrin A and calbistrin C were produced under these culture conditions (Fig. 1b). Previous studies of calbistrins have shown that the [M + H]^+^ ions are not observed in the mass spectra due to extensive water losses [7, 8] and we therefore searched for the presence of the sodium ion adducts, [M + Na]^+^, for the two compounds. Inspection of the chromatograms for the WT revealed the presence of the calbistrin A [M + Na]^+^ m/z of 563.2623 (calculated 563.2621, mass error of 0.355 ppm) eluting at 9.7 min, and fragment ions corresponding to neutral losses of one, two and three water molecules for calbistrin A (Additional file 1: Additional Information 1A), and the calbistrin C [M + Na]^+^ m/z of 565.2776 (calculated 565.2777, mass error of 0.177 ppm) eluting at 9.9 min (Additional file 1: Additional Information 1B). These adduct- and fragmentation patterns assisted the establishment of monoisotopic masses and indicated molecular formulas of C_31_H_40_O_8_ and C_31_H_42_O_8_, corresponding to calbistrin A and C, respectively. The identity of calbistrin A was confirmed by comparison of the UV spectrum and the MS/HRMS fragmentation pattern to that of an in-house reference standard for calbistrin A (Additional file 1: Additional Information 1C-D). Tentative identification of calbistrin C was based on comparison of it MS/HRMS fragmentation pattern to that of calbistrin A (Additional file 1: Additional Information 3F-I).

The UHPLC-HRMS analysis of the wild type grown on CM (Fig. 1b) also revealed [M + Na]^+^ parent ions that corresponded to the three compounds decumbenone A (two isomers eluting at 6.02 and 6.50 min), decumbenone B (eluting at 6.25 min), and decumbenone C (two isomers eluting at 4.4 and 5.05 min). As the decumbenones all have the same polyketide backbone length and decalin moiety as the calbistrins (Fig. 1a), we hypothesized that they are intermediates in, or byproducts of, calbistrin biosynthesis. The identity of these compounds could not be definitively confirmed due to the lack of reference standards, however, the fragmentation patterns for the putative decumbenone A-C compounds were in good agreement with the fragmentation patterns of calbistrin A and C (Additional file 1: Additional Information 3).

Further inspection of the WT chromatogram revealed the presence of two peaks (eluting at 5.9 and 6.7 min in Fig. 1b) that had a composition of C_15_H_20_O_5_, based on HRMS, which corresponds to the composition of the linear dioic acid moiety of calbistrins and therefore also could be related to calbistrin biosynthesis. This hypothesis was further strengthened by the finding that MS/HRMS fragments of these compounds were identical to several MS/HRMS fragments observed upon fragmentation of calbistrin A and C (Additional file 1: Additional Information 4). Furthermore inspection of the MS/HRMS data of the putative dioic acid moieties showed neutral losses of CO (at RT 5.8 min: fragment ions of m/z 199.1112 and m/z 171.1161 give a difference of 27.9951, at RT 6.9 min: fragment ions of m/z 199.1120 and m/z 171.1166 give a difference of 27.9954; theoretical mass CO = 27.9949) and sequential losses of 1C fragments, supporting the predicted molecular features (Additional file 1: Additional Information 2 and 4). Finally, the most abundant peak (5.9 min) had the same distinct UV spectrum as the calbistrins with absorption maxima at 345 nm (Additional file 1: Additional Information 4) (the peak at 6.9 min was too small for detection of UV spectrum). One should note that calbistrins are known to feature several different cis–trans isomers of the linear dioic acid moiety, e.g. calbistrin A constist excluselively of trans conformations while calbistrin B and D include a single cis conformation at various positions [8]. These cis–trans transitions were shown to be induced by light exposure which also occurred during extraction [18].

### Comparative genomics of *P. decumbens* identifies a PKS putatively involved in calbistrin biosynthesis

The genome of *P. decumbens* (IBT11843), a member of the *Penicillium* subgenus *Aspergilloides* clade, was recently sequenced [10]. To narrow down the candidates for the calbistrin PKSs, a comparative genomics analysis with two distantly related known calbistrin producers was conducted. *A. aculeatus* has been reported to produce calbistrin A and C [19], and *A. versicolor* has been reported to produce versiol [20], which has a related structure to the decalin part of calbistrin A. Putative PKSs in *A. aculeatus and A. versicolor* were identified similar as described for *P. decumbens*, yielding 26 and 27 putative PKSs respectively.

Additionally, several further fungal PKS and PKS-NRPS-like biosynthetic systems have been reported to produce decalin containing metabolites, e.g. lovastatin in *A. terreus*, compactin in *Penicillium brevicompactum*, solanapyrone in *Alternaria solani* [21], equisetin/fusarisetin A in *Fusarium heterosporum* and *Fusarium sp.* FN080326 [22, 23] and myceliothermophin in *Myceliophthora thermophile* [24]. The enzymatic basis for decalin formation in these systems is however not identical and falls into at least three distinct groups: (1) PKS/PKS-NRPS based cycloadditions as seen in LovB and MlcA [25], (2) post-PKS bifunctional oxidases/alderases, such as Sol5 in *Alternatria solani* [21], (3) post-PKS monofunctional alderases of diverse evolutionary origin such as the Fsa2 from the fusaristatin/equisetin pathways [23], and MycB (AEO57198) from the myceliothermophin E pathway. Nonetheless, to test how putative orthologous PKSs could be related to the known decalin forming PKSs, we decided to include the KS-AT domains of MlcA, LovB, EqxS, Sol1, Fsa1 and MycA in the phylogenetic analysis.

**Fig. 2:**
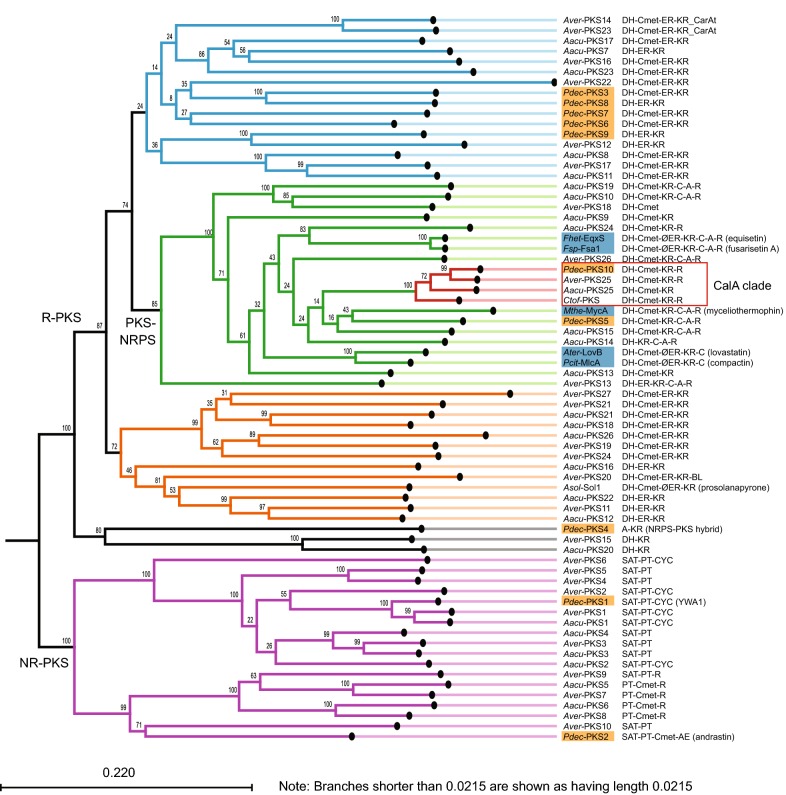
Neighbour joining tree of KS-AT domains from *P. decumbens*, *A. aculeatus* and *A. versicolor* PKSs. The four-membered clade with putative calbistrin-forming PKSs is highlighted with a red square. Known decalin forming PKSs are highlighted with blue background. Abbreviations: Species: *Pdec*: *P. decumbens* (highlighted in orange); *Aacu*: *A. aculeatus*; *Aver*: *A. versicolor*; *Fhet*: *F. heterosporum*; *Fsp*: *Fusarium* sp. FN080326; *Mthe*: *Myceliophthora thermophile*; *Ater*: *A. terreus*; *Pcit*: *P. citrinum.* Enzymatic domain: DH: dehydratase; Cmet: C-methyl transferase; ER: enoylreductase; ØER: dysfunctional ER; KR: ketoreductase; C: condensation; A: Adenylation; R: terminal reductase; TE: thioesterase; CarAt: carnetine acyltransferase; BL: beta lactamase; AE: acetylesterase; PT: product template; SAT: Starter acyltransferase, CYC: cyclase

Subsequently, the KS domains were aligned using the Smith-Waterman algorithm and a neighbour joining tree was constructed to identify putative orthologous enzymes across the three species (Fig. 2). The analysis showed that five of the six known decalin-forming PKSs (highlighted with blue in Fig. 2) clustered within a single well supported clade (bootstrap of 85%) of PKS-NRPS hybrids. This clade includes true PKS-NRPS hybrids, and hybrids where part or the whole NRPS portion has been lost. PdecPKS10 proved to be closest related to the myceliothermophin forming PKS-NRPS MycA from *M. thermophile*, then the equisetin forming PKS-NRPSs from *Fusarium* sp. and lastly the statin forming PKS-NRPSs LovB and MlcA. The close association with known decalin forming PKSs supports the hypothesis that PdecPKS10 is responsible for formation of the decalin portion of calbistrin. This hypothesis was further supported by the fact that KS domains from the partially reducing PKSs AspacPKS25 and AspvePKS25 clustered also with PdecPKS10, having an average identity of 76%. These three PKSs were all predicted to include a β-ketosynthase (KS), an acyltransferase (AT), a dehydratase (DH), a methyltransferase (MT), a ketoreductase (KR), an acyl carrier protein (ACP), and a terminal reductase (R) domain.

Further analysis of the neighbour joining tree showed that only one additional clade included members of all three species, suggesting orthologous PKSs (Fig. 2). This clade included KS domains of three non-reducing PKSs (PdecPKS1, AspacPKS1 and AspvePKS1) which showed very high sequence similarities (average of 76%) to the wA PKS from *P. rubens* and therefore likely are responsible for producing YWA-based pigments in the respective species.

The comparative genomics analysis of PKSs in the three known calbistrin producers did not reveal any obvious candidates for the second PKS predicted to be responsible for synthesizing the linear dioic acid portion of calbistrin.

### Deletion of the *pdecPKS10* gene demonstrates involvement of the PKS in calbistrin production

To test the proposed association between PdecPKS10 activity and calbistrin formation we adapted a transformation and targeted genetic engineering system recently developed for *P. chrysogenum* (also known as *Penicillium rubens* Wisconsin 54-1255) [26] for use in *P. decumbens* to delete the PKS encoding gene PENDEC_c013G00595.

This protocol resulted in sufficiently high gene editing efficiencies to generate several clones for characterization of *calA* and also *calB* and *calC* in *P. decumbens* (Additional file 1: Additional Information 5). Initial screening of the generated transformants at the gene locus by colony PCR and sequencing of clones displaying a size shift of the PCR product, indicating that excision of the entire genomic DNA region framed by the used protospacers was the most prevalent recombination event (Additional file 1: Additional Information 13 to 15), followed by some cases where parts from the AMA-plasmid had integrated albeit no microhomology sequences were detected between the inserts and genomic locus. Surprisingly, neither of the analyzed transformants contained simpler short indel mutations as would be expected following incorrect repair of a single cut event by the NHEJ pathway (Additional file 1: Additional Information 13 to 15). Based on our observations, the success rate of future experiments can perhaps be increased by adding a target-specific donor DNA repair templates although this would increase the experimental preparation effort we sought to reduce here.

Analysis of the *PdecPKS10* mutant (∆PKS) showed that the production of calbistrin was completely abolished whereas production of unrelated compounds, such as andrastin C, remained unaffected (Figs. 1b, 6). These results confirmed that PdecPKS10 is essential for the biosynthesis of the calbistrins. Interestingly, the masses of the putative related metabolites, decumbenones and the putative linear moiety, also disappeared in the PKS deletion strains. This shows that these metabolites are involved in calbistrin biosynthesis, as hypothesized.

### Defining the putative gene clusters boundaries by gene synteny analysis and transcriptomics data

**Fig. 3:**
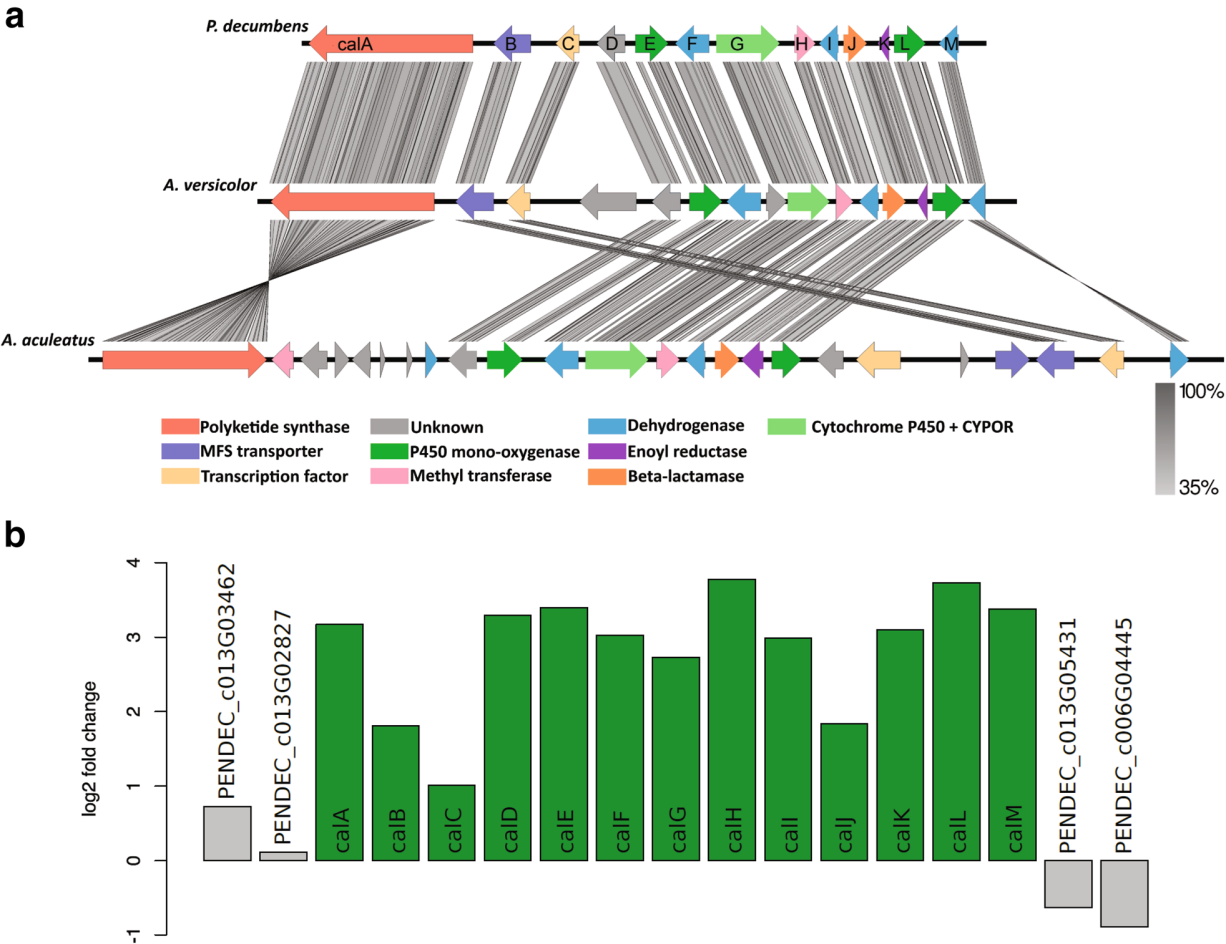
Expression and gene synteny in calbistrin cluster. **a** Synteny analysis of putative gene clusters in *P. decumbens*, *A. aculeatus* and *A. versicolor.* Figure made with EasyFig [46]. **b** Transcription analysis of calbistrin cluster in wild-type strain under calbistrin producing vs non-producing conditions. Log2 fold change for read counts in complex medium (inductive) over synthetic medium (non-inductive)

A more detailed bioinformatic analysis of the *PdecPKS10* locus revealed that several of the adjacent genes encoded proteins with putative tailoring enzyme functions were presumably relevant for the biosynthesis of calbistrins. To determine the boundaries of this putative gene cluster, we performed a synteny analysis of the respective contigs containing *PdecPKS10*, *AspacPKS25* and *AspvePKS25*. The analysis clearly showed conserved regions around the predicted PKS genes (Fig. 3a, trimmed to clusters for clarity) covering 10 predicted genes in *P. decumbens* (spanning a region of 35 kb) that displayed sequence similarity with a region containing 14 predicted genes upstream of the PKS in *A. versicolor* and 14 predicted genes in *A. aculeatus* downstream of the PKS. The identified conserved region in *P. decumbens* was continuous, while in *A. versicolor* the syntenic region was disrupted by a single gene that did not show homology with regions in the two other species. The putative cluster in *A. aculeatus* included two regions with no homology to regions in the two other species consisting of one region of seven adjacent genes and a second region of four adjacent genes.

The *P. decumbens* gene cluster included several regions that displayed high sequence similarity to the other two species but which lacked predicted genes, suggesting a less successful gene calling in *P. decumbens*. Guided by the detected homology, we used FGENESH as an alternative gene prediction and predicted three additional genes, which resulted in a total of 13 putative genes in the *P. decumbens* conserved region, named *calA*-*calM*. The proteins encoded by these genes all showed identities of > 75% and > 50% at amino acid level with the enzymes encoded the conserved regions in *A. versicolor* and *A. aculeatus,* respectively (Table 1). At least one conserved functional domain was found in 12 out of the 13 predicted proteins, while none was found in CalD (Table 1). Ten of the proteins included predicted enzymatic functionality which would support a function as tailoring enzymes in secondary metabolite biosynthesis. The predicted enzymes were two cytochrome P450 monooxygenases (CalE and CalL), a bifunctional CYP-P450 monooxygease fused with a CYP-P450 reductase domain (CalG), three dehydrogenases (CalF, CalI and CalM), a methyltransferase (CalH), an enoyl reductase (CalK) and a beta lactamase (CalJ). In addition, two of the proteins included domains indicative of a MFS transporter (CalB) and a GAL4-like Zn(II)2Cys6 transcription factor (CalC), respectively. Analysis of the proteins encoded upstream of the PKS in *A. aculeatus* revealed two proteins (a putative methyl transferase and a short-chain dehydrogenase/reductase) that could be part of a biosynthetic gene cluster. However, these genes are present in multiple copies in the genomes of the two other species and hence likely not involved in calbistrin biosynthesis (Additional file 1: Additional information 6).

**Table 1:** Putative proteins within the calbistrin cluster in *P. decumbens*

Name	*P. decumbens locus*	Size (aa)	BLASTP *Aver*	%I	BLASTP *Aacu*	%I	Conserved domain and notes	E-value
CalA	PENDEC_c013G00595	2910	OJJ08178.1	85.3	XP_020058113.1	78.1	PKS: KS, AT, ACP, DH, Cmet, KR, R Note: similar to MlcA PKS	0.0
CalB	PENDEC_c013G07044	562	OJJ08177.1	84.9	XP_020058136.1	79.4	TIGR00711, drug resistance transporter, Note: similar to MlcE MFS pump	4.1E−40
CalC	PENDEC_c013G06298	426	OJJ08176.1	74.6	XP_020058137.1	51.9	smart00066, GAL4-like Zn(II)2Cys6 DNA-binding domain	3.3E−05
CalD	PENDEC_c013G04601	494	OJJ08174.1	89.6	XP_020058121.1	76.1	No putative conserved domains detected.	
CalE	PENDEC_c013G04259	494	OJJ08173.1	77.2	XP_020058122.1	49.8	pfam00067, Cytochrome P450 Note: similarity to MlcC monooxygenase	1.8E−36
CalF	PENDEC_c013G03789	575	OJJ08172.1	85.4	XP_020058123.1	75.4	COG0277, FAD/FMN-containing dehydrogenase Note: similar to the bifunctional Sol5 flavin-dependent oxidase and alderase from *Alternaria solani*	2.7E−22
CalG	n/a	1056	OJJ08171.1	84.9	XP_020058124.1	72.8	pfam00067, Cytochrome P450, + CYPOR Bifunctional: N-term cytochrome P450 and C-term cytochrome P450 reductase domains	2.5E−78
CalH	PENDEC_c013G02261	273	OJJ08170.1	82.6	XP_020058125.1	61.2	pfam08242, SAM dependent methyltransferase Note: similarity to C-MET domain found in HR-PKSs: FUM1, EasB, LepA, ApdA and AzaB	1.6E−20
CalI	PENDEC_c013G00477	383	OJJ08169.1	82.7	XP_020058126.1	72.4	PRK06196, oxidoreductase (dehydrogenase)	1.1E−75
CalJ	n/a	418	OJJ08168.1	82.1	XP_020058127.1	68.5	pfam00144, Beta-lactamase (putative acyltransferase) Note: similar to MlcH acyltransferase	1.7E−33
CalK	PENDEC_c013G03312	194	OJJ08167.1	83.0	XP_020058128.1	67.0	cd08249, enoyl reductase like Note: similar to MlcG ER	3.5E−110
CalL	PENDEC_c013G00617	568	OJJ08166.1	88.1	XP_020058129.1	78.2	pfam00067, Cytochrome P450	5.7E−23
CalM	n/a	304	OJJ08165.1	89.8	XP_020058138.1	78.3	PRK06180, short chain dehydrogenase	1.5E−67

The gene names *calA*–*calM* were defined in this study. The PENDEC_XXXXX accession numbers are as in the original publication of the genome, except for *calG*, *calJ* and *calM.* These new gene models were constructed using Softberry FGENESH supported with homologous genes in *A. versicolor* (*Aver*) and *A. aculeatus* (*Aacu*) (see Additional file 1: additional information 16 for protein sequences of *P. decumbens* CalG, CalJ and CalM proteins). Putative homologues of each of the *P. decumbens* CAL protein in *A. aculeatus* and *A. versicolor* were identify by BLASTP are here presented with accession umber, along with % identity at amino acid level (%I) along with the predicted conserved domains found in the protein and E-value for this prediction

Moreover, a BLASTP analysis with the *P. decumbens* CalA-CalM proteins revealed that CalA-CalM showed high identities not only with proteins from *A. versicolor* and *A. aculeatus*, but also with several proteins from *Colletotrichum tofieldiae* and *Colletotrichum chlorophyti.* An additional gene synteny analysis with scaffold 170 (accession LFIV01000170.1) of *C. tofieldiae* revealed the presence of a similar cluster in *C. tofieldiae,* but several rearrangements in the order of the genes (Additional file 1: Additional information 7). All predicted proteins in the calbistrin cluster, except for CalJ, were found to have a homologue in the *C. tofieldiae* cluster.

The putative calbistrin cluster was further analysed for co-expression with the aim of identifying the boundaries of the cluster. Transcriptomics data (RNA-seq) of *P. decumbens* grown in liquid CM, supporting calbistrin production, was compared with that of *P. decumbens* grown in liquid DM where calbistrin is not produced (unpublished). The resulting log2 fold change plot showed that all 13 predicted genes in the putative cluster were upregulated in CM compared to DM (Fig. 3b and Additional file 1: additional information 8), while neighbouring genes did not show differential expression. This further strengthened the hypothesis of the proposed boundaries of the cluster.

### The transcription factor CalC is required for calbistrin production

**Fig. 4:**
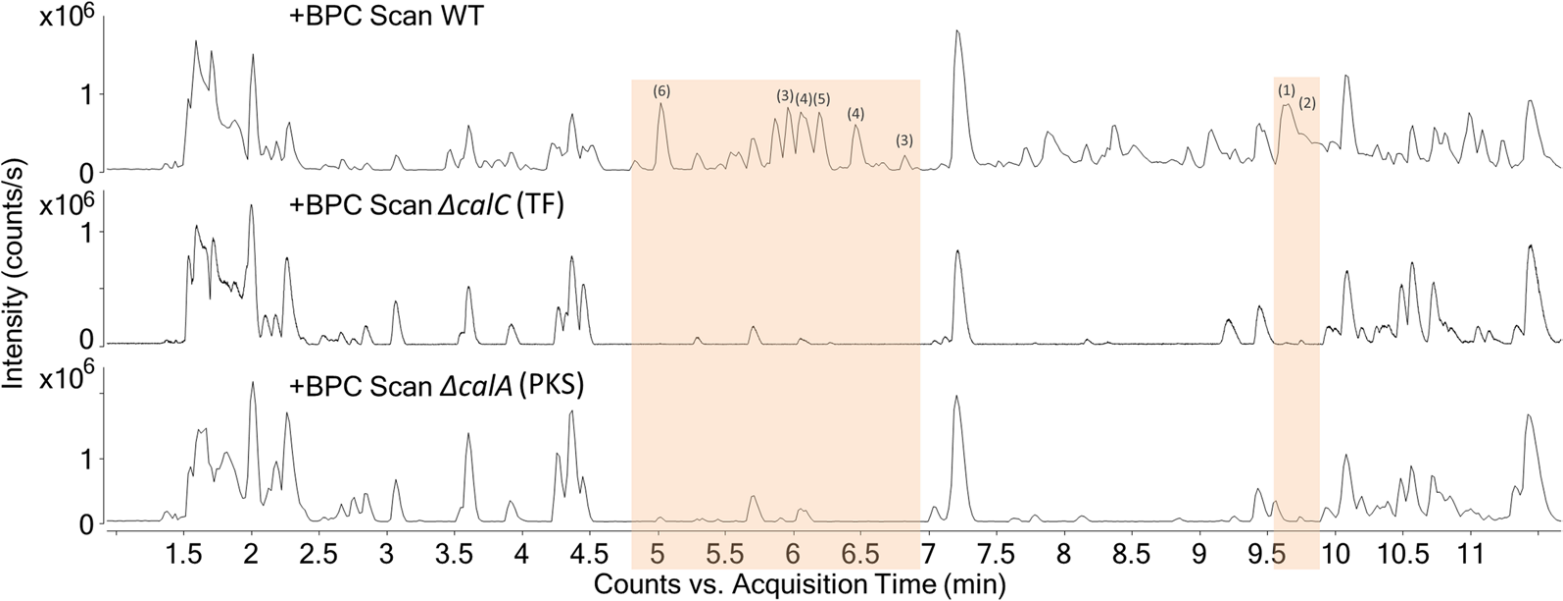
Comparison of UHPLC-HRMS results of *P. decumbens* ∆TF and *P. decumbens* ∆PKS compared to WT. Base peak chromatograms (BPCs) of *P. decumbens* WT, *P. decumbens* ∆TF and ∆PKS

**Fig. 5:**
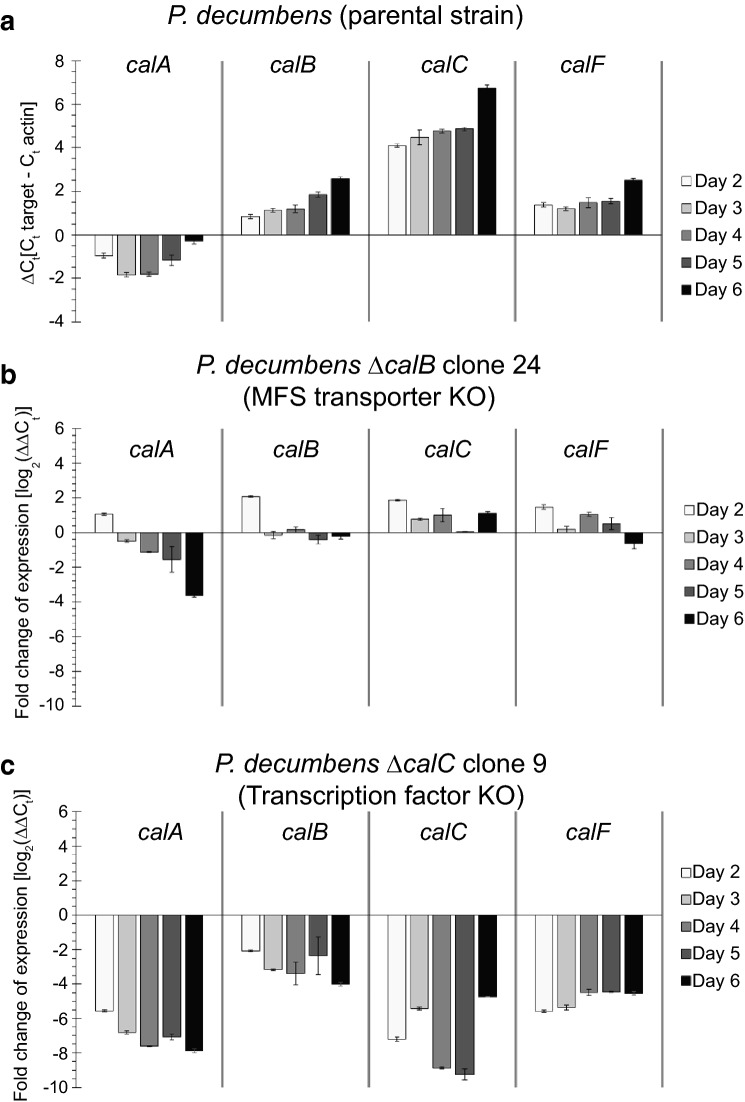
Gene expression profiles of *P. decumbens* parental and loss-of-function strains grown in liquid CM. **a** Gene expression of *calA*, *calB*, *calC* and *calF* in wild type *P. decumbens* strain relative to actin. Data are averages from two independent grown flasks analyzed in two technical duplicates. **b** Gene expression profile of *calA*, *calB*, *calC* and *calF* in *P. decumbens* ∆*calB*—loss-of-function strain relative to the wild type strain. **c** Gene expression profile of *calA*, *calB*, *calC* and *calF* in *P. decumbens* ∆*calC*—loss-of-function strain relative to the parental strain

One of the encoded proteins in the *P. decumbens* cluster, CalC, was predicted to include an N-terminal located GAL4-like Zn(II)2Cys6 binuclear zinc cluster DNA-binding domain and a C-terminally located fungal specific transcription factor domain, a domain architecture typically found in secondary metabolite gene cluster specific transcription factor (TF) [27]. Targeted deletion of the gene *calC,* using CRISPR/Cas9, and metabolic profiling of the resulting mutant (∆*calC*) revealed a similar chemical profile to that of the PKS deletion mutant: a complete disappearance of calbistrins and related compounds (Fig. 4). The deletion did not affect the production of non-related compounds, such as andrastin C, suggesting that the CalC TF is only regulating the transcription of a limited number of genes rather than secondary metabolism in general, as observed for other PKS cluster specific TFs. The function of CalC as an activating transcription factor controlling the calbistrin cluster was further supported by a qPCR based expression analysis of the *calA*, *calB*, and *calF* genes, which showed that deletion of CalC resulted in a significant downregulation of the tree analysed genes (*calA*, *calB* and *calF*) in the cluster (Fig. 5).

### The MFS transporter CalB is involved in calbistrin export

**Fig. 6:**
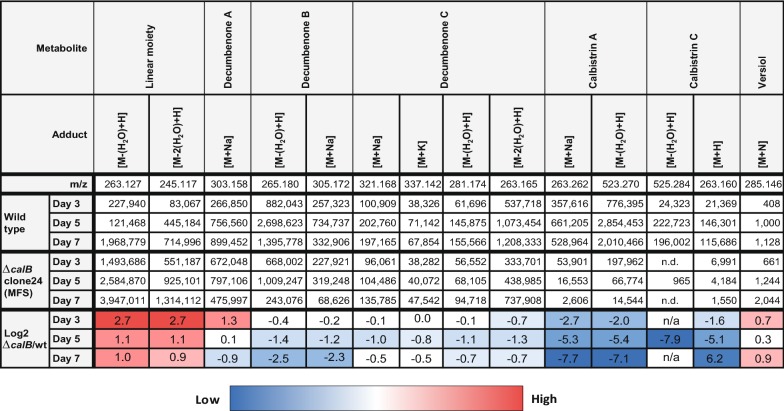
Heat map of tracked masses in MS analysis of *P. decumbens* strains grown in liquid CM. To account for growth differences between strains, peak areas were corrected by CDW. Changes in corrected peak areas of calbistrin A and related compounds in the KO strain of *calB* were compared to the wild type strain. Reduced abundancy of calbistrin A suggests that calB is required for efficient excretion of calbistrins

Targeted deletion of *calB*, encoding a predicted major facilitator superfamily transporter and HPLC-HRMS based profiling of the extracellular secondary metabolites produced in CM broth after day 5 and 7 showed an almost complete absence of calbistrin A and calbistrin C, a decreased abundancy of decumbenone A, B and C to 20–60% of the wild type levels and increased amounts of the linear moiety (Fig. 6). This suggests that CalB is involved in export of calbistrin A and related metabolites containing a decalin moiety. Analysis of the transcriptional response of *calA*, *calC* and *calF* in the ∆*calB* background indicated an earlier decrease in transcription for *calA* and a moderate log2 fold change (log2FC) in expression of 1 for *calC* and *calF* (Fig. 5b), suggesting that the lack of calbistrin export and consequently a putative intracellular increase did not strongly impact the expression of these 2 genes. The need for active transport is likely due to the dioic acid moiety that increases the molecule size of calbistrin and causes changes in surface charge distribution, reducing the likelihood of a partial non calB-dependent transport or passive leakage out of the cells across the membrane as observed with remaining amounts of decumbenones and the linear moiety in the broth after transporter deletion.

### Search for the second PKS required for calbistrin production

**Fig. 7:**
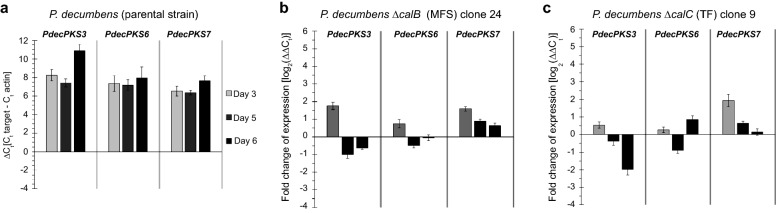
Expression analysis of putative candidate PKSs for production of the linear moiety of calbistrins. **a** Gene expression relative to actin for 3 putative PKS capable of performing a C-methylation in the *P. decumbens* wild type strain. **b** and **c** Gene expression changes in loss-of-function mutants of ∆*calB* and ∆*calC*, respectively, compared to the wild type strain. No complete absence of expression was detected in either of the deletion strains, suggesting that none of the PKSs are transcriptionally controlled by molecules from the calbistrin pathway

Calbistrin is predicted to consist of two individually formed polyketide chains [8] that differ both in their length and decoration pattern, requiring the activity of two independent polyketide synthases as seen in statin biosynthesis. The high similarity between the KS domain of CalA and other known decalin producing PKS systems strongly indicate that CalA is responsible for biosynthesis of the decalin moiety, while the linear moiety must be produced by a second unknown PKS encoded by a gene located elsewhere in the genome. However, surprisingly deletion of *calA* did not only result in the inability to produce the decalin containing metabolites (calbistrin A, B, decumbenone A, B, C), but also hampered production of the linear dioic acid moiety, suggesting an inaction of the unknown PKS. Similar shutdown of entire biosynthetic pathways has been observed for other secondary metabolite cluster and pathways, e.g. bikaverin biosynthesis in several *Fusarium* species [28], where deletion of structural genes can result in the transcriptional down regulation of the remaining genes in the cluster. The molecular basis for such down regulations is currently unknown, but may be utilized to identify unknown components of a biosynthetic system. Therefore, we performed a qPCR expression analysis of the three PKS candidates (*PdecPKS3*, *PdecPKS6*, *PdecPKS7*) for the unknown dioic acid forming activity in the TF deletion strain (∆*calC*) and in the MFS deletion strain (∆*calB*) which was still able to produce all intermediates but performed poorly in export of calbistrin A and B. The analysis showed that the expression of the three PKS encoding genes did not change dramatically, less than two fold, in neither of the two strains (Fig. 7) suggesting that they are most likely are not responsible for forming the linear moiety. Targeted deletion of *pdecPKS6* and chemical analysis of the mycelium and agar-plug extracts confirmed this conclusion for this gene as no change in calbistrin-associated secondary metabolites were detected (data not shown). However, it cannot be conclusively excluded that *PdecPKS7* and *PdecPKS3* based on the presented data and it is possible that formation of the dioic acid occurs in an alternative fashion independent of PKSs.

## Discussion

Comparative genomics analysis of three species producing the bioactive secondary metabolite calbistrin led to the identification of a partly reducing PKS (Fig. 2), that proved to be involved in calbistrin production in *P. decumbens* (Fig. 1). Further comparative analysis identified a region consisting of 13 genes that was shared between the three species. In *P. decumbens* this was a continuous region, while the syntenic region was disrupted in *A. versicolor* by a single gene and in *A. aculeatus* by two regions of seven and four genes, respectively (Fig. 3). In all cases, antiSMASH predicted larger clusters than what was predicted via the synteny based comparative analysis (34 vs. 13 genes in P*. decumbens*, 19 vs. 14 in *A. versicolor* and 33 vs. 23 in *A. aculeatus*). However, the smaller cluster predicted by the synteny analysis was supported by RNA-seq data in *P. decumbens* which showed co-expression of the 13 genes.

Deletion of the PKS encoding gene *pdecPKS10* in *P. decumbens* eliminated calbistrin production proving its involvement in the biosynthesis of calbistrin. However, calbistrin consists of two polyketides, one decalin containing 14 carbon backbone and one linear 12 carbon backbone, and is therefore predicted to be synthesized by two polyketide synthases [8]. Besides the absence of the calbistrins and the putative decalin containing precursors decumbenone A-C, formation of the putative dioic acid moiety was also absent in the *PdecPKS10* deletion strain, producing a situation that made it impossible to conclusively determine whether PdecPKS10 is responsible for synthesis of the decalin or the dioic acid moiety of calbistrin.

However, based on the high sequence identity of the PdecPKS10 KS-AT domains to that of other known decalin forming PKSs, such as MlcA, LovB, EqxS, Sol1 and Fsa1, we suggest that CalA is responsible for forming the decalin moiety (Fig. 2). This hypothesis is strengthened by the reductase (R) domain predicted at the C-terminal end of CalA. The decalin containing decumbenones have a terminal aldehyde instead of the carboxylic acid usually obtained from a classical thioesterase (TE) based release mechanism, and the R domain in CalA could be responsible for reducing the thioester bond to release the product as the observed aldehyde. Resembling the product release mechanism reported for the PKS-NRPS hybrids MycB and EqxS/Fsa1 that both includes terminal reductase domains resulting in the formation of terminal aldehyde groups in the products [23, 24]. A situation that differs markedly from the LovB PKS that does not include TE or R domains, but instead dependent on the trans-acting thioesterase LovG for product release [29].

Calbistrin includes fully reduced ketide units and one would hence expect the involved PKS to include an enoylreductase (ER) domain, however, the identified CalA lacks this domain. Nonetheless, one gene within the calbistrin cluster, *calK*, is predicted to have an ER conserved domain. The involvement of a trans-acting ER is also seen in the lovastatin/compactin, myceliotheramophin and equisetin biosynthesis, where the PKSs contains an inactive ER domain and reduction of the backbone is catalysed by an trans-acting accessory enzyme, LovC in lovastatin biosynthesis [30, 31]. As CalK belongs to the same family of enoylreductases as LovC (conserved protein domain family accession cd08249: enoyl_reductase_like) it could potentially be responsible for carrying out this reductive step on the growing calbistrin polyketide chain.

The enzymatic basis for [4 + 2] cycloaddition that leads to formation of decalin structures differs significantly between fungal systems and while the statin-forming PKS have been shown to catalyse the reaction themselves [31], other systems depend on trans-acting alderases that act on the polyketide chains following release from the PKS. A search for homologs of the monofunctional alderases Fsa2 and MycB in *P. decumbens* did not return any significant hits, however a search for the bifunctional Sol5 revealed CalF as a significant hit. Sol5 from *A. solani* is a bifunctional flavin-dependent oxidase and Diels-Alderase responsible for catalysing the cycloaddition in solanapyrone [21]. Based on the high level of similarity between CalA and CalF to the enzymes in the salanapyrone pathways and we hence hypothesise that the decalin part of calbistrins is formed via a similar mechanism. The decalin polyketide backbone includes two C-methyl groups, at C7 and C11 in backbone, of which the C7 positions is similarly to what is seen in compactin, where it is known to be added by the PKSs C-methyltransferase domain. A candidate for adding the methyl group at C11, if not done by CalA, is CalH that resembles the C-methyltransferase domains found in the FUM1 (fumonisin), EasB (Emericellamide), LepA (leporins), ApdA (Aspyridones) and AzaB (Azaphilone) PKSs (Table 1).

The genes found upstream of the PKS *calA* gene encodes several tailoring enzymes that potentially could be involved in the modification of the decalin polyketide product (Table 1). This includes three P450 monooxygenases (CalE, CalG and CalL), of which one might be responsible for the introduction of the extra hydroxyl group attached to the backbone of the decalin moiety, at position C9 in the backbone, that allows for attachment of the linear moiety. One tailoring enzyme activity that is expected to be involved in biosynthesis of calbistrin is an acyltransferase for connecting the two polyketide synthase products, such as seen in lovastatin biosynthesis, where the acyltransferase LovD is involved in transferring the polyketide chain from the PKS LovF to the finished polyketide product from the PKS LovB [32]. Blasting of the LovD protein sequence against the predicted *P. decumbens* proteins resulted in the identification of four proteins (E-value below 1 × 10E−38), of which CalJ had the highest level of sequence identity, of 33%, to LovD of the four hits. CalJ was initially predicted to be an acyltransferase, as the conserved domain with the highest score was a beta lactamase domain. However, this was also the case for LovD which previously has been experimentally proved to act as an acyltransferase. Similarly, it has been demonstrated that EstB, a protein related to beta-lactamases, lacked ß-lactamase activity but instead act as a acyltransferase in the bacteria *Burkholderia gladioli* [33].

The calbistrin cluster identified in this study potentially encodes many of the enzymatic activities predicted to be required for de novo synthesis of calbistrin. However, explaining synthesis of the linear moiety remains a challenge. The first obvious hypothesis for a second PKS responsible for the biosynthesis of the linear moiety would be the presence of another PKS in the close genomic vicinity of *calA* (*PdecPKS10*), similarly to the situation described for the PKSs involved in lovastatin and compactin formation. However, no other PKS was predicted on *P. decumbens* scaffold 13, which suggest that the calbistrin pathway may be encoded by several different loci in the genome. The *P. decumbens* genome is only predicted to encode a total of ten PKSs, of which one was predicted to be responsible for YWA synthesis (Pdec-PKS19), one for andrastin A synthesis (PdecPKS2), and one was found to be involved in calbistrin synthesis in this study (PdecPKS10). The structure of both the decalin and the linear moieties suggest that they undergo a C-methylation of the backbone chain during synthesis, with the decalin possessing two methyl groups and three are present in the linear structure. The *P. decumbens* genome included other than PdecPKS10, three putative PKSs with a predicted C-methylation domain: PdecPKS6, PdecPKS7 and PdecPKS3. Another option is that the C-methylation is catalysed by a post-PKS tailoring enzyme. The gene located on the genome next to *PdecPKS4* was annotated as a putative methyl transferase, and thus could possibly perform a post-PKS C-methylation reaction. Based on our data, we can exclude PdecPKS6 as being responsible for formation of the linear moiety based on targeted deletion. Further investigation of calbistrin biosynthesis could therefore focus on the deletion of *PdecPKS3*, *PdecPKS4* and PdecPKS7, and evaluate their role in biosynthesis of the linear moiety.

Deletion of the predicted transcription factor encoding gene *calC* resulted in the abolishment of the production of calbistrin and its related metabolites, proving involvement of CalC in calbistrin formation (Fig. 4) by regulating expression of *PdecPKS10.* Comparison of the *calC* mutant metabolite profile revealed that it was very similar to that of the PKS deletion strain, suggesting that the transcription factor regulates the cluster, and does not act as a global regulator. Indeed, GAL4-like type of transcription factors are the most common type of in-cluster pathway regulators in fungi [27]. To further investigate the influence of the transcription factor on the calbistrin cluster, expression of several genes in the cluster was compared between the wild-type and the ∆TF strains (Fig. 5). The observation that the final product calbistrin, the decalin intermediates as well as the linear dioic acid intermediates disappeared similarly upon deletion of the PKS and the transcription factor is interesting.

One speculation could be the existence of a negative feedback mechanism triggered by the absence of the decalin intermediates results in the shut-down of the biosynthetic pathway of the linear intermediate, either at enzymatic or gene expression level. Alternatively the lack of the decalin metabolite in the cell results in a situation where the activity of the PKS that forms the linear polyketide is inhibited as it is unable to unload its formed product due to a lack of the decalin reaction partner. Another possibility for the synthesis of the dioic (dicarboxylic) acid would be the oxydation of a free long-chain fatty acid to a ω-hydroxy acid via a cytochrome P450 monoxygenases [34] and subsequent oxidation via alcohol and aldehyde dehydrogenases [35]. Indeed, the putative cluster contains three genes with a putative P450 monoxygenase function (CalE, CalG and CalL), however, this scenario is very speculative as the dioic acid moiety of calbistrin is branched and desaturated, requiring intensive enzymatic activity to source this molecule via the free fatty acid biosynthetic pathway. In contrast, one could argument that the premature release product of CalA could be the starter unit for a P450 monoxygenase and does not undergo cyclysation in this case.

Deletion of the predicted MFS transporter gene *calB* resulted in a strong decrease (log2FC of − 6.2 to − 7.7 on day 5) of extracellular calbistrin A and C levels and a modereate decrease (log2FC of − 0.5 to − 2.3 on day 5) of extracellular decumbenone A, B and C, suggesting that calB is involved in export of both decumbenones and calbistrin, however the latter molecules do seem to get exported to some extent via other less specific transport routes, as their level did not decrease as strongly as the level of calbistrin A and C. Similar observations were made for export of andrastatin A in *Penicillium roqueforti* [36] and bikaverin in *Fusarium fujikuroi* [28] when their respective transporter was downregulated or deleted, respectively. Although we did not perform analysis of intracellular accumulation of calbistrin A and C, we can conclude from our expression data that a possible feedback on the transcript level due to accumulation of calbistrin A, C or its pathway intermediates did not occur for *calA*, *calC* and *calF* as the expression levels only changed modestly, making it unlikely that complete abolishment of expression takes place. However, it still remains a possibility that enzymes not analyzed for their expression in this study show a stronger response or the inhibition takes place on the enzyme level.

Future studies could also look beyond calbistrins as molecule class and investigate the mode of action and benefits for the producers of the related decumbenones which were shown to inhibit melanisation of *Magnaporthe grisea* [37] and stimulating the germination of agricultural plants [38]. As our study also identified a potential calbistrin cluster in the root endophyte *Colletotrichum tofieldiae* which supports growth of *Arabidopsis thalina* under low phosphate conditions [39], it might be worth to investigate the production and role of decumbenones and calbistrins in these interactions and wether they are valuable for fending off other soil-thriving fungi or promoting growth of the host plant.

## Conclusions

This study identified a 13-membered gene cluster in *P. decumbens* required for biosynthesis of the structurally interesting polyketide calbistrin. Targeted deletion of three of the identified genes, namely *PdecPKS12* (*calA*), *calB* and *calC*, proved their involvement in the formation of calbistrins, as a polyketide synthase, a pump and a positively acting pathway specific transcription factor, respectively. The identified Cal-cluster encode many of the required enzyme types predicted to be essential for de novo calbistrin biosynthesis, however, the enzyme(s) responsible for formation of the linear moiety remains elusive and further work is hence needed to allow for the future construction of a high yielding calbistrin cell factory.

## Methods

### Strains and media

*Penicillium decumbens* strain IBT11843 was obtained from and is available at the IBT culture collection (Department of Biotechnology and Biomedicine, Technical University of Denmark).

For chemical analysis, strains were grown either on liquid or solidified Czapek yeast autolysate medium (CM) containing (30 g/l sucrose, 5 g/l yeast extract, 3 g/l NaNO_3_, 1.0 g/L K_2_HPO_4_, 0.5 g/l MgSO_4_·7 H_2_O, 0.5 g/l KCl, 0.01 g/l FeSO_4_,·7 H_2_O, 20 g/l agar and 1.0 mL trace metal solution containing 0.1 g/l ZnSO_4_·7H2O and 0.05 g/L CuSO_4_·5 H_2_O, the pH was adjusted to 6.2 with NaOH.

For transcriptome data referred to in this study, cultivations were performed in CM and defined medium (DM) as described previously [40]. For preparing protopalasts for transformation of *P. decumbens*, YGG medium was used for cultivation as described previously [41].

### Bioinformatic analysis

Genome sequences from *P. decumbens* IBT 11843 (accession MDYL00000000) [10], *A. aculeatus* ATCC 16872 (accession MRCK00000000.1) [42] and *A. versicolor* CBS 583.65 (accession MRBN00000000) [42] were obtained from GenBank.

To identify biosynthetic gene clusters (BGCs) in *P. decumbens*, the genome was analysed via the AntiSMASH (v.3.0.4) server, resulting in the prediction of in total 22 putative BGCs, of which nine included PKS encoding genes. A previous analysis of 24 genome sequenced *Penicillium* species, showed that these in average encoded 17.2 PKS BGCs [10]. The low number of identified PKS encoding genes in *P. decumbens* prompted us to perform an additional BLAST based search for PKS encoding genes that may have been missed in the first round of automated analysis. The manual analysis was performed using the β-ketosynthase (KS) domain from the YWA producing PKS (accession XP_002568608) from *Penicillium rubens* Wisconsin 54-1255 as query in a TBLASTN search against a database containing the translations of the *P. decumbens* whole genome sequence in all six open reading frames and a BLASTP search against a database containing all predicted proteins in the *P. decumbens* genome. Full length protein sequences for hits with an e-value below 1e-6 in the BLASTP analysis were retrieved and annotated using the NCBI Conserved Domain Database [43]. This resulted in the identification of one additional highly reducing PKS, bringing the total to five highly reducing PKSs (HR-PKSs), one partially reducing PKS (PR-PKS), two non-reducing PKSs (NR-PKSs), and two partially reducing PKS-nonribosomal peptide synthetase hybrids (PR-PKS-NRPS).

CLC main Workbench version 7 (QIAGEN Bioinformatics) was used for local BLAST analysis, protein alignment and neighbor joining tree creation. The amino acid sequences of the PKSs for all organisms were trimmed to the KS-AT domains, which are the only universal domains of PKSs and have previously been shown to be a good evolutionary determinant [10, 44]. Phylogenetic trees were exported to the iTOL v3 tool for manual annotation and visualization [45]. Gene predictions in *P. decumbens* were performed using FGENESH (Softberry). Functional conserved domains in the translated protein sequences were predicted using Conserved Domain Search (NCBI). Analysis of syntenic regions was done using the python application Easyfig [46].

RNA-seq data were obtained from Jens Nielsen’s lab at Chalmers University (Nielsen et al., unpublished). Raw reads were mapped to the *P. decumbens* reference genome (accession MDYL00000000) using TopHat2 (v. 2.0.9) [47], and gene read counts were quantified using FeatureCount [48], both with default parameters. Differential expression analysis was computed for complex medium relative to defined medium using DESeq 2 [49].

### Fungal transformation and gene disruption in *P. decumbens*

Protoplasts of *P. decumbens* were prepared 48 h after inoculation of 5 × 10^5^ spores/ml in YGG medium using the methods and media described previously [41] with the following modifications: we reduced the incubation time in glucanex solution (30 mg/ml in KC Buffer) to 75 min, as longer incubation reduced the number of recovered colonies (For an overview of conducted transformations for this publication, see Additional file 1: Additional information 5).

To establish which dominant selection markers can be used for *P. decumbens*, protoplast were initially plated on [41] containing either 0.1% acetamide (Sigma Aldrich, NL) as the sole nitrogen source or 40 mM sodium nitrate and one of the following selection agents: 50 µg/ml phleomycin (Invivogen, USA) or 1.2 µg/ml terbinafine (Terbinafine Hydrochloride, Sigma Aldrich, NL).

In contrast to a lack of inhibition on acetamide plates (due to activity of host acetamidase genes), robust inhibition of growth was observed on plates with phleomycin and terbinafine. Low inhibitory concentration of terbinafine have been previously reported by Sigl et al. [50] for *Penicillium chrysogenum*. As terbinafine acts as an inhibitor of squalene epoxidase in a broad range of fungi and is also convenient from an economic point of view, we used the MoClo modular cloning system [51] to construct an *ergA* overexpression cassette utilizing the widely used pgpdA promoter from *Aspergillus nidulans* (Additional file 1: Additional information 9) and the squalene epoxidase *ergA* from *Penicillium chrysogenum* to build pCP-AMA-ergA, which was utilized when deleting *calC* and *calB*.

For protospacer selection, sgRNA synthesis and RNP delivery we used the methods described in [26] with an additional filtering for highly active protospacers using sgRNA scorer 2.0 [52]. For selection of protoplasts competent in taking up DNA (and presumably other macromolecules such as RNPs), either 3.0 µg pJAK-109 [26] or pCP-AMA-ergA were co-transformed along with RNPs and protoplasts were plated on protoplast recovery plates supplemented with phleomycin or terbinafine and 40 mM sodium nitrate. Plates were incubated for up to 7 days at 25 °C to allow recovery of transformants and formation of colonies.

Colonies were screened by colony PCR using Phire Green 2x Mastermix (Thermo Scientific, The Netherlands) and initial anaylsis of band size shifts on 1% agarose gels. To determine length and location of insertions or deletions (Additional file 1: Additional information 12–15) Sanger sequencing (Macrogen, The Netherlands) of PCR products was performed.

To loose AMA-plasmids obtained during transformation, spores were harvested and diluted out on nonselective R-Agar [41] followed by colony PCR. This procedure was repeated twice. A list of all sgRNAs and primers used in this study can be found in Additional file 1: Additional information 10 and 11, respectively.

### qPCR analysis of calA, B, C and M in *P. decumbens*

For qPCR analysis of the calbistrin cluster genes in *P. decumbens*, we choose a single ∆*calB* and ∆*calC* clone and 3 biological replicates of the parental strain. 1 ml of a spore solution (1x10^6^ spores/ml) was used for inoculation of 25 ml liquid CM in 100 ml shake flasks. Cultures were grown for 7 days at 25 °C and 200 rpm. Mycelium for RNA extraction was separated from 5 ml broth by filtration, washed once with 2 volumes of ice-cold H_2_O and 100–200 mg wet biomass were mixed with 1 ml Trizol reagent (Thermo Fisher Scientific, The Netherlands), transferred to screw-cap tubes containing glass beads (diameter 0.75–1 mm) and stored at − 80 °C until RNA isolation. Mycelium was disrupted with a FastPrep FP120 system (Qbiogene, France) and total RNA was isolated using the Direct-zol RNA MiniPrep Kit (Zymo Research, USA). For cDNA synthesis, 1500 ng total RNA were reverse transcribed using the Maxima H Minus cDNA Synthesis Master Mix (Life Technologies, The Netherlands) in a volume of 20 µl. Samples were diluted with 80 µl MQ-H_2_O and 4 µl of this cDNA were used as input for qPCR in a final volume of 25 µl. As master mix for qPCR, the SensiMix SYBR Hi‐ROX (Bioline Reagents, England) was used. All runs were performed on a MiniOpticon system (Bio‐Rad). The following conditions were employed for amplification: 95 °C for 10 min, followed by 40 cycles of 95 °C for 15 s, 60 °C for 30 s and 72 °C for 30 s, following an acquisition step. Raw ct data were exported and analysis of relative gene expression was performed with the 2 − ΔΔ*CT* method [53]. The expression analysis was performed with two technical duplicate per biological sample. The γ‐actin gene (PENDEC_c001G04327) was used as internal standard for data normalization. The primers used for qPCR of *calA* (PENDEC_c013G00595), *calB* (PENDEC_c013G07044), *calC* (PENDEC_c013G06298), *calF* (PENDEC_c013G03789) and γ‐actin are listed in (Additional file 1: Additional information 11).

### Chemical analysis

For solid cultures, three agar plugs were sampled from one colony and 1.0 ml of extraction solvent, isopropanol:ethylacetate (1:3) containing 1% formic acid, was added. After ultra-sonication for 1 h the extract was transferred to a clean vial, evaporated to dryness and dissolved in 100 µl methanol. After centrifugation for 5 min the supernatant was directly used for chemical analysis.

Secondary metabolite analysis of solid culture samples was achieved by ultra-high performance liquid chromatography-diode array detection-quadrupole time of flight mass spectrometry (UHPLC-DAD-QTOFMS) on an Agilent 1290 UHPLC system (Agilent Technologies, Torrance, CA) coupled to an Agilent 6545 QTOF equipped with an electrospray ionization (ESI) source. True tandem MS/HRMS spectra were obtained at fixed collision-induced dissociation (CID) energies of 10, 20, and 40 eV [54] and matched to the available reference standards of calbistrin A and andrastin C.

For analysis of cultures grown in liquid CM, broth and mycelium were separated by centrifugation for 10 min at 14,000 g, followed by filtration of the clarified broth over 0.2 µm PTFE syringe filters (VRW, The Netherlands). The obtained filtrate was directly used for analysis or frozen at − 20 °C. For analysis of liquid culture samples, high performance liquid chromatography electrospray-ionization high–resolution mass spectrometry (HPLC-ESI-HRMS) was conducted on an Accella1250 UPLC system coupled to an Orbitrap Exactive (Thermo Fisher Scientific, The Netherlands) with a scan range of m/z 100–1600. A sample of 10 μL was injected onto a Shim-pack XR-ODS C18 column (75 mm × 3.0 ID, 2.2 μm) (Shimadzu, Japan) kept at 40 °C and operated at a flow rate of 300 μL/min. Separation of compounds was achieved with the following solvents (A: 100% MQ-H_2_O, B: 100% Acetonitrile, and C: 2% formic in MQ-H_2_O being constantly added at 5% to protonate molecules). After injection of sample, column was run for 5 min with isocratic flow at 5% B, following a linear gradient for 25 min reaching 95% B, remaining constant at 95% B for 5 min and equilibrating the column with initial conditions of 5% B for 5 min before injection of the next sample. Each sample was analyzed in technical duplicate. Total ion chromatograms and areas of m/z of interest were generated and processed using MassHunter (Agilent) and XCalibur (ThermoFisher) with a m/z error below 1 ppm for all molecules referred to in this study.

## Additional file

**Additional file 1.** Additional figures, tables and pictures for “Identification of the decumbenone biosynthetic gene cluster *in Penicillium decumbens* and the importance for production of calbistrin”.

## References

[1] van den Berg M, Albang R, Albermann K, Badger JH, Daran JM, Driessen AJM (2008). Genome sequencing and analysis of the filamentous fungus *Penicillium chrysogenum*. Nat Biotechnol.

[2] Pickens LB, Tang Y, Chooi Y-H (2011). Metabolic engineering for the production of natural products. Annu Rev Chem Biomol Eng.

[3] Nielsen JC, Nielsen J (2017). Development of fungal cell factories for the production of secondary metabolites: linking genomics and metabolism. Synth Syst Biotechnol.

[4] O’Connor SE (2015). Engineering of secondary metabolism. Annu Rev Genet.

[5] Jackson M, Karwowski JP, Humphrey PE, Kohl WL, Barlow GJ, Tanaka SK (1993). Calbistrins, novel antifungal agents produced by *Penicillium restrictum*. I. Production, taxonomy of the producing organism and biological activity. J Antibiot (Tokyo).

[6] Bertizal FK, Dombrowski AW, Helms GL, Horn WS, Jones ETT, Koupal L, et al. JPH08134059 (A)—Cholesterol lowering agent. 1991.

[7] Bladt TT, Durr C, Knudsen PB, Kildgaard S, Frisvad JC, Gotfredsen CH (2013). Bio-activity and dereplication-based discovery of ophiobolins and other fungal secondary metabolites targeting leukemia cells. Molecules.

[8] Brill GM, Chen RH, Rasmussen RR, Whittern DN, McAlpine JB (1993). Calbistrins, novel antifungal agents produced by *Penicillium restrictum* II. Isolation and elucidation of structure. J Antibiot (Tokyo).

[9] Stewart M, Capon RJ, Lacey E, Tennant S, Gill JH (2005). Calbistrin E and two other new metabolites from an Australian isolate of *Penicillium striatisporum*. J Nat Prod.

[10] Nielsen JC, Grijseels S, Prigent S, Ji B, Dainat J, Nielsen KF (2017). Global analysis of biosynthetic gene clusters reveals vast potential of secondary metabolite production in *Penicillium* species. Nat Microbiol.

[11] Fujii Y, Asahara M, Ichinoe M, Nakajima H (2002). Fungal melanin inhibitor and related compounds from *Penicillium decumbens*. Phytochemistry.

[12] Endo A, Monacolin K (1979). new hypocholesteroleic agent produced by a *Monascus species*. J Antibiot (Tokyo).

[13] Alberts AW, Chen J, Kuron G, Hunt V, Huff J, Hoffman C (1980). Mevinolin: a highly potent competitive inhibitor of hydroxymethylglutaryl-coenzyme A reductase and a cholesterol-lowering agent. Proc Natl Acad Sci U S A.

[14] Brown BAG, Srnale TC, Pharmaceuticals B, Park B, King TJ, Hasenkamp R (1976). Crystal and molecular structure of compactin, a new antifungal metabolite from *Penicillium brevicompactum*. JCS Perkin.

[15] Frisvad JC, Filtenborg O (1989). Terverticillate *Penicillia*: chemotaxonomy and mycotoxin production. Mycologia.

[16] Endo A, Kuroda M, Tsujita Y (1976). ML-236A, ML-236B, and ML-236C, new inhibitors of cholesterogensis produced by *Penicillium citrinum*. J Antibiot (Tokyo).

[17] Auclair K, Sutherland A, Kennedy J, Witter DJ, Van Den Heever JP, Hutchinson CR (2000). Lovastatin nonaketide synthase catalyzes an intramolecular diels—alder reaction of a substrate analogue. J Am Chem Soc.

[18] Horn WS, Bierilo KK, Bills GF, Dombrowski AW, Helms GL, Jones ET (1993). Characterization of the light- and base-mediated instability of calbistrin A. J Nat Prod.

[19] Petersen LM, Hoeck C, Frisvad JC, Gotfredsen CH, Larsen TO (2014). Dereplication guided discovery of secondary metabolites of mixed biosynthetic origin from *Aspergillus aculeatus*. Molecules.

[20] Fukuyama K, Hamasaki T, Hatsuda Y, Hamasaki T, Nakagomi T, Fukuyama K (1978). Structure and absolute configuration of versiol, a metabolite from *Aspergillus versicolor*. J Chem Soc Perkin Trans 2.

[21] Kasahara K, Miyamoto T, Fujimoto T, Oguri H, Tokiwano T, Oikawa H (2010). Solanapyrone synthase, a possible Diels–Alderase and iterative type I polyketide synthase encoded in a biosynthetic gene cluster from *Alternaria solani*. ChemBioChem.

[22] Kakule TB, Sardar D, Lin Z, Schmidt EW (2013). Two related pyrrolidinedione synthetase loci in *Fusarium heterosporum* ATCC 74349 produce divergent metabolites. ACS Chem Biol.

[23] Kato N, Nogawa T, Hirota H, Jang J-H, Takahashi S, Ahn JS (2015). A new enzyme involved in the control of the stereochemistry in the decalin formation during equisetin biosynthesis. Biochem Biophys Res Commun.

[24] Li L, Yu P, Tang M-C, Zou Y, Gao S-S, Hung Y-S (2016). Biochemical characterization of a eukaryotic decalin-forming Diels–Alderase. J Am Chem Soc.

[25] Campbell CD, Vederas JC (2010). Biosynthesis of lovastatin and related metabolites formed by fungal iterative PKS enzymes. Biopolymers.

[26] Pohl C, Kiel JAKW, Driessen AJM, Bovenberg RAL, Nygård Y (2016). CRISPR/Cas9 based genome editing of *Penicillium chrysogenum*. ACS Synth Biol.

[27] Yin W, Keller NP (2011). Transcriptional regulatory elements in fungal secondary metabolism. J Microbiol.

[28] Wiemann P, Willmann A, Straeten M, Kleigrewe K, Beyer M, Humpf HU (2009). Biosynthesis of the red pigment bikaverin in *Fusarium fujikuroi*: genes, their function and regulation. Mol Microbiol.

[29] Xu W, Chooi YH, Choi JW, Li S, Vederas JC, Da Silva NA (2013). LovG: the thioesterase required for dihydromonacolin L release and lovastatin nonaketide synthase turnover in lovastatin biosynthesis. Angew Chemie Int Ed.

[30] Auclair K, Kennedy J, Hutchinson CR, Vederas JC (2001). Conversion of cyclic nonaketides to lovastatin and compactin by a lovC deficient mutant of *Aspergillus terreus*. Bioorganic Med Chem Lett.

[31] Kennedy J, Auclair K, Kendrew SG, Park C, Vederas JC, Hutchinson CR (1999). Modulation of polyketide synthase activity by accessory proteins during lovastatin biosynthesis. Science (80-).

[32] Xie X, Meehan MJ, Xu W, Dorrestein PC, Tang Y (2009). Acyltransferase mediated polyketide release from a fungal megasynthase. J Am Chem Soc.

[33] Petersen EI, Valinger G, Solkner B, Stubenrauch G, Schwab H (2001). A novel esterase from *Burkholderia gladioli* which shows high deacetylation activity on cephalosporins is related to beta-lactamases and DD-peptidases. J Biotechnol.

[34] Durairaj P, Malla S, Nadarajan SP, Lee P-G, Jung E, Park HH, Kim B-G, Yun H (2015). Fungal cytochrome P450 monooxygenases of *Fusarium oxysporum* for the synthesis of ω-hydroxy fatty acids in engineered *Saccharomyces cerevisiae*. Microb Cell Fact.

[35] Bowen CH, Bonin J, Kogler A, Barba-Ostria C, Zhang F (2016). Engineering *Escherichia coli* for conversion of glucose to medium- chain ω-hydroxy fatty acids and α, ω-dicarboxylic acids. ACS Synth Biol.

[36] Rojas-Aedo JF, Gil-Durán C, Del-Cid A, Valdés N, Álamos P, Vaca I, García-Rico RO, Levicán G, Tello M, Chávez R (2017). The biosynthetic gene cluster for andrastin A in *Penicillium roqueforti*. Front Microbiol.

[37] Fujiia Y, Asahara M, Ichinoe M, Nakajima H (2002). Fungal melanin inhibitor and related compounds from *Penicillium decumbens*. Phytochemistry.

[38] Anisimov MM, Chaikina EL, Afiyatullov SS, Zhuravleva OI, Klykov AG, Kraskovskaja NA, Aminin DL (2012). Decumbenones A–C from marine fungus *Aspergillus sulphureus* as stimulators of the initial stages of development of agricultural plants. Agric Sci.

[39] Hiruma K, Gerlach N, Sacristán S, Nakano RT, Hacquard S, Kracher B, Neumann U, Ramírez D, Bucher M, O’Connell RJ, Schulze-Lefert P (2016). Root endophyte *Colletotrichum tofieldiae* confers plant fitness benefits that are phosphate status dependent. Cell.

[40] Grijseels S, Nielsen JC, Nielsen J, Larsen TO, Frisvad JC, Fog Nielsen K (2017). Physiological characterization of secondary metabolite producing *Penicillium* cell factories. Fungal Biol Biotechnol.

[41] Kovalchuk A, Weber SS, Nijland JG, Bovenberg RAL, Driessen AJM, Bolton M, Thomma B (2012). Fungal ABC transporter deletion and localization analysis. Plant fungal pathogens methods in molecular biology.

[42] de Vries RP, Riley R, Wiebenga A, Aguilar-Osorio G, Amillis S, Uchima CA (2017). Comparative genomics reveals high biological diversity and specific adaptations in the industrially and medically important fungal genus *Aspergillus*. Genome Biol.

[43] Marchler-Bauer A, Derbyshire MK, Gonzales NR, Lu S, Chitsaz F, Geer LY (2015). CDD: NCBI’s conserved domain database. Nucleic Acids Res.

[44] Kroken S, Glass NL, Taylor JW, Yoder OC, Turgeon BG (2003). Phylogenomic analysis of type I polyketide synthase genes in pathogenic and saprobic ascomycetes. Proc Natl Acad Sci.

[45] Letunic I, Bork P (2016). Interactive tree of life (iTOL) v3: an online tool for the display and annotation of phylogenetic and other trees. Nucleic Acids Res.

[46] Sullivan MJ, Petty NK, Beatson SA (2011). Easyfig: a genome comparison visualizer. Bioinformatics.

[47] Kim D, Pertea G, Trapnell C, Pimentel H, Kelley R, Salzberg SL (2013). TopHat2: accurate alignment of transcriptomes in the presence of insertions, deletions and gene fusions. Genome Biol.

[48] Liao Y, Smyth GK, Shi W (2014). FeatureCounts: an efficient general purpose program for assigning sequence reads to genomic features. Bioinformatics.

[49] Love MI, Huber W, Anders S (2014). Moderated estimation of fold change and dispersion for RNA-seq data with DESeq2. Genome Biol.

[50] Sigl C, Handler M, Sprenger G, Kurnsteiner H, Zadra I (2010). A novel homologous dominant selection marker for genetic transformation of *Penicillium chrysogenum*: overexpression of squalene epoxidase-encoding ergA. J Biotechnol.

[51] Weber E, Engler C, Gruetzner R, Werner S, Marillonnet S (2011). A modular cloning system for standardized assembly of multigene constructs. PLoS One..

[52] Chari R, Yeo NC, Chavez A, Church GM (2017). SgRNA scorer 2.0: a species-independent model to predict CRISPR/Cas9 activity. ACS Synth Biol.

[53] Livak KJ, Schmittgen TD (2001). Analysis of relative gene expression data using real-time quantitative PCR and the 2(-Delta Delta C(T)) method. Methods.

[54] Kildgaard S, Mansson M, Dosen I, Klitgaard A, Frisvad JC, Larsen TO (2014). Accurate dereplication of bioactive secondary metabolites from marine-derived fungi by UHPLC-DAD-QTOFMS and a MS/HRMS library. Mar Drugs.

