# Supplementary material for: Identification of the decumbenone biosynthetic gene cluster in Penicillium decumbens and the importance for production of calbistrin
Source: Fungal Biol Biotechnol. 2018 Dec 19;5:18. doi: 10.1186/s40694-018-0063-4 (PMC6299560; doi:10.1186/s40694-018-0063-4)
Supplement: Supplementary file 1 — Additional file 1. Additional figures, tables and pictures for “Identification of the decumbenone biosynthetic gene cluster in Penicillium decumbens and the importance for production of calbistrin”. [file 40694_2018_63_MOESM1_ESM.docx]

# Additional information

# Identification of the decumbenone biosynthetic gene cluster *in Penicillium decumbens* and the importance for production of calbistrin

Sietske Grijseels^1+^, Carsten Pohl^2+^, Jens Christian Nielsen^3^, Zahida Wasil^1^, Yvonne Nygård^2^, Jens Nielsen^3,4^, Jens C. Frisvad^1^, Kristian Fog Nielsen^1^, Mhairi Workman^1^, Thomas Ostenfeld Larsen^1^, Arnold J.M. Driessen^2^, Rasmus John Normand Frandsen^1^*

+ These authors contributed equally

^1^Department of Biotechnology and Biomedicine, Technical University of Denmark, DK2800 Kgs. Lyngby, Denmark

^2^Molecular Microbiology, Groningen Biomolecular Sciences and Biotechnology Institute, University of Groningen, 9747 AG Groningen, the Netherlands

^3^Department of Biology and Biological Engineering, Chalmers University of Technology, SE412 96 Gothenburg, Sweden

^4^Novo Nordisk Foundation Center for Biosustainability, Technical University of Denmark, DK2800 Kgs. Lyngby, Denmark

* Corresponding author.

Additional information 1. A) Full scan of calbistrin A in the *P. decumbens* extract, B) Full scan of calbistrin C in the *P. decumbens* extract, C) MS/HRMS of calbistrin A in the *P. decumbens* extract, D) MS/HRMS of calbistrin A in the reference standard. The true tandem MS/HRMS spectra are obtained at 20 eV with an Agilent 6545 QTOF.


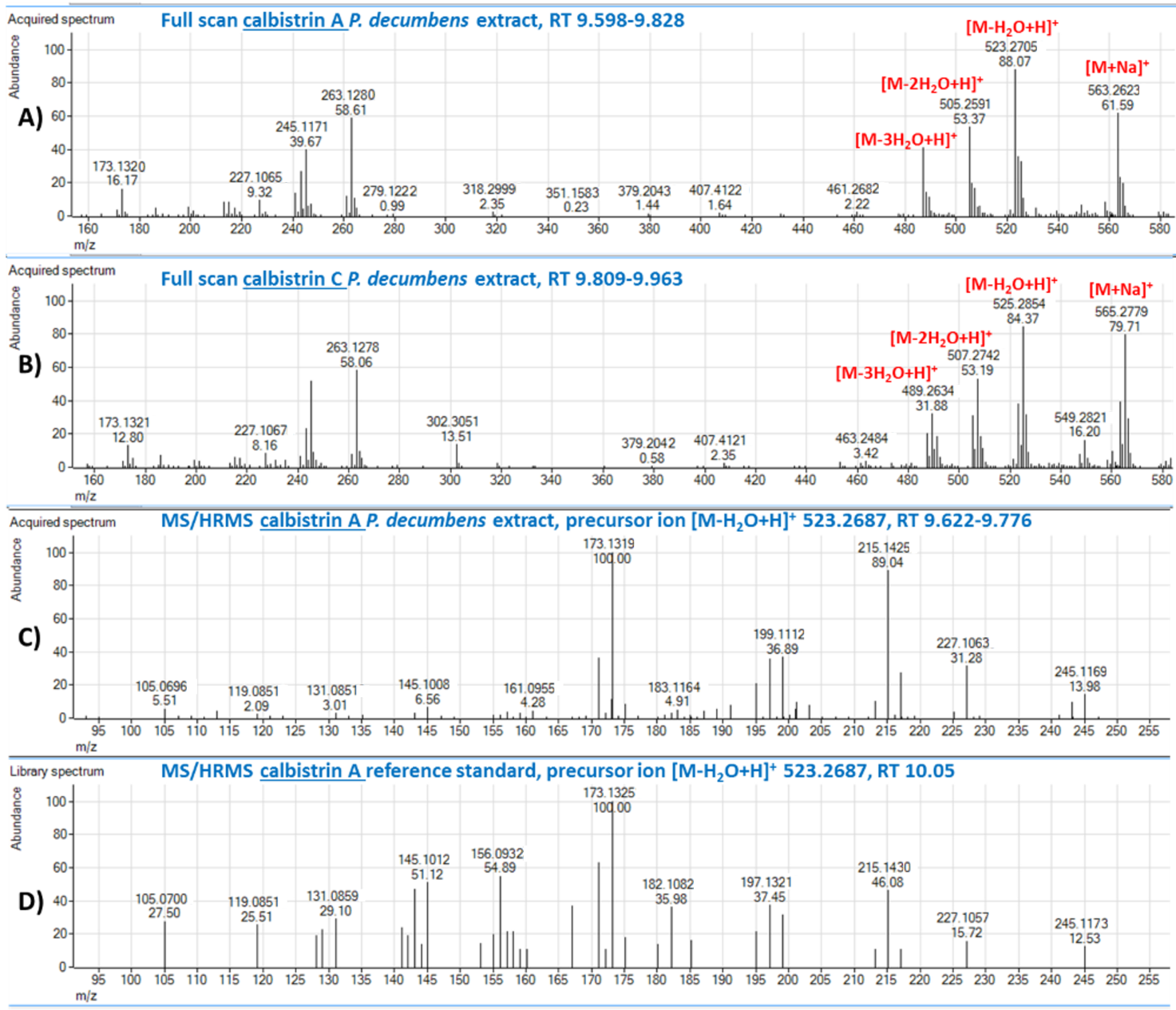


Additional information 2. Molecular formula, name, retention time (RT) and m/z of observed adducts in calbistrin and related metabolites.

For several compounds there is more than one retention time, these are likely stereo isomers of the same compound. The difference between the theoretical m/z of the given compounds and the measured is given as Δppm in parenthesis after the m/z value.

| **No.** | **Name** | **Molecular formula** | **RT** | **[M-2(H2O)+H]**  **(∆ppm)** | **[M-(H2O)+H]**  **(∆ppm)** | **[M+H]**  **(∆ppm)** | **[M+Na]**  **(∆ppm)** | **[M+K]**  **(∆ppm)** |
| --- | --- | --- | --- | --- | --- | --- | --- | --- |
| 1 | Decumbenone C | C_16_H_26_O_5_ | 4.40 | 263.1643 (0.4) | 281.1742 (1.8) |  | 321.1670 (0.6) | 337.1401 (3.3) |
|  |  |  | 5.05 | 263.1654 (4. 6) | 281.1744 (1.0) |  | 321.1677 (1.6) | 337.1416 (1.2) |
| 2 | Linear moiety | C_15_H_20_O_5_ | 5.90 | 245.1177 (2.0) | 263.1286 (3.0) |  | 303.1205 (0.7) |  |
|  |  |  | 6.85 | 245.1173 (0.4) | 263.1281 (1.1) |  | 303.1205 (-1.0) |  |
| 3 | Decumbenone A | C_16_H_24_O_4_ | 6.02 | 245.1536 (0.0) | 263.1662 (7.6) |  | 303.1572 (0.0) |  |
|  |  |  | 6.50 | 245.1538 (0.8) | 263.1640 (-0.8) | 281.1751 (1.4) | 303.1575 (2.6) | 319.1331 (7.8) |
| 4 | Decumbenone B | C_16_H_26_O_4_ | 6.25 | 247.1697 (1.6) | 265.1806 (3.0) |  | 305.1720 (1.0) |  |
| 5 | Calbistrin A | C_31_H_40_O_8_ | 9.70 | 505.2591 (1.2) | 523.2705 (2.9) |  | 563.2623 (1.4) |  |
|  | (calbistrin B |  | 10.50 | 505.2587 (0.4) | 523.2700 (1.9) |  | 563.2614 (0.2) |  |
|  | or D) |  | 11.00 | 505.2586 (0.2) | 523.2696 (1.2) |  | 563.2623 (1.4) |  |
| 6 | Calbistrin C | C_31_H_42_O_8_ | 9.9 | 507.2738 (0.6) | 525.2847 (0.0) |  | 565.2776 (0.7) |  |

**Additional information 3. Tentative identification of decumbenone A, decumbenone B, and decumbenone C by MS/HRMS fragmentation analysis. A)** MS/HRMS decumbenone C isomer 1 (4.4 min), **B)** MS/HRMS decumbenone C isomer 2 (5.0 min), **C)** MS/HRMS decumbenone A isomer 1 (6.5 min), **D)** MS/HRMS decumbenone A isomer 2 (6.0 min), **E)** MS/HRMS decumbenone B (6.2 min), **F)** MS/HRMS calbistrin A isomer 1 (9.6 min), **G)** MS/HRMS calbistrin A isomer 2 (10.5 min), **H)** MS/HRMS calbistrin A isomer 3 (11.0 min), and **I)** MS/HRMS calbistrin C (9.8 min). Fragment ions shared between the compounds indicated with red underscore. The retention time of the individual compound refers to Figure 1B. 1


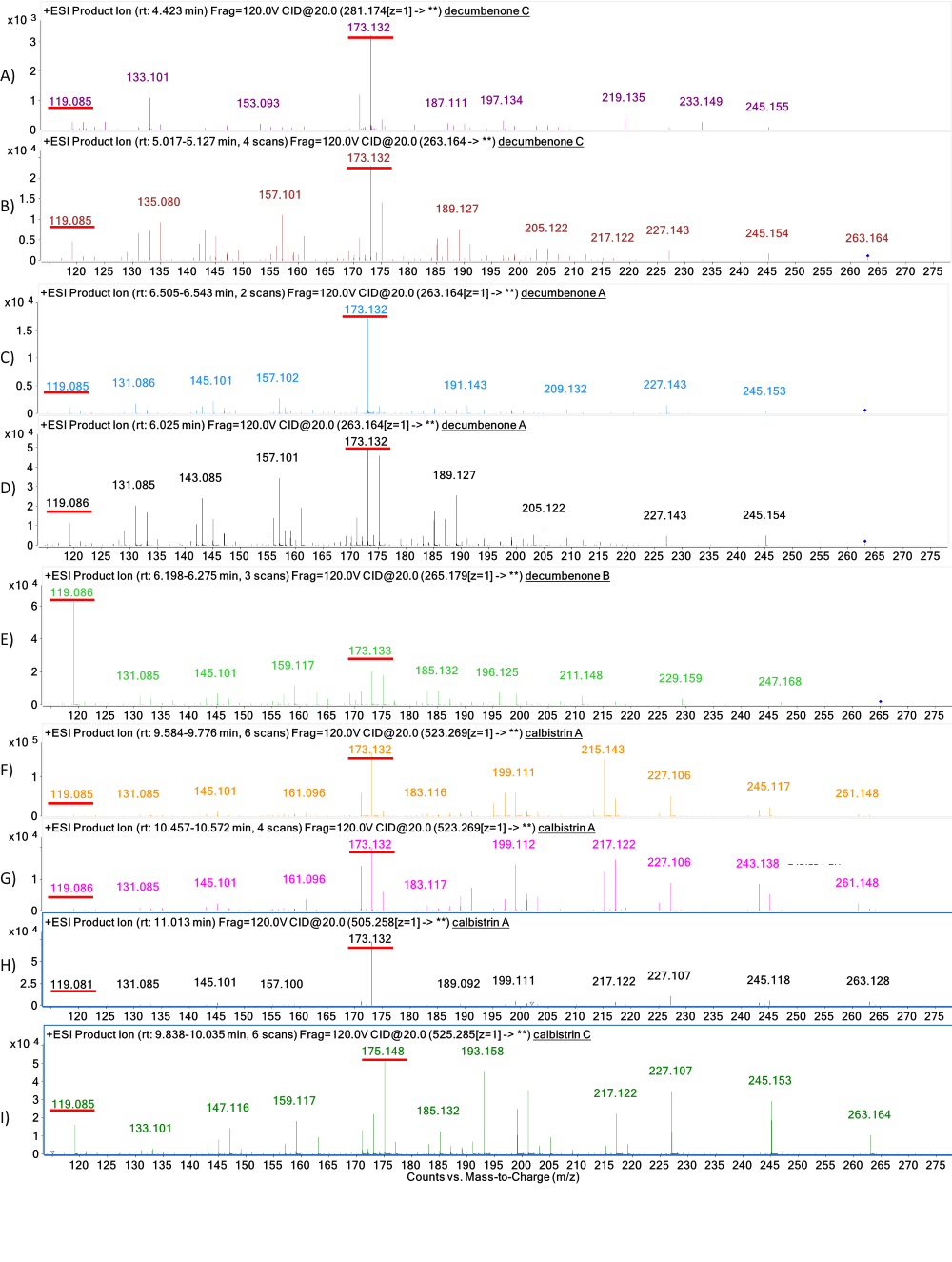


Additional information 4. Tentative identification of dioic acid moiety. Upper panel: A) Fragmentation analysis (MS/HRMS) of calbistrin A, B) Fragmentation analysis (MS/HRMS) of the tentatively identified dioic acid isomer 1 (5.9 min), C) Fragmentation analysis (MS/HRMS) of the tentatively identified dioic acid isomer 2 (6.9 min). Fragment ions shared between calbistrin A and the tentative dioic acid isomers indicated with blue underscore. Lower panel: UV spectrum of calbistrin A (red) and tentative dioic acid moiety at 5.83 min (black) in *P. decumbens* extract. Abundance of isomer 2 was too low for UV spectrum detection.

| 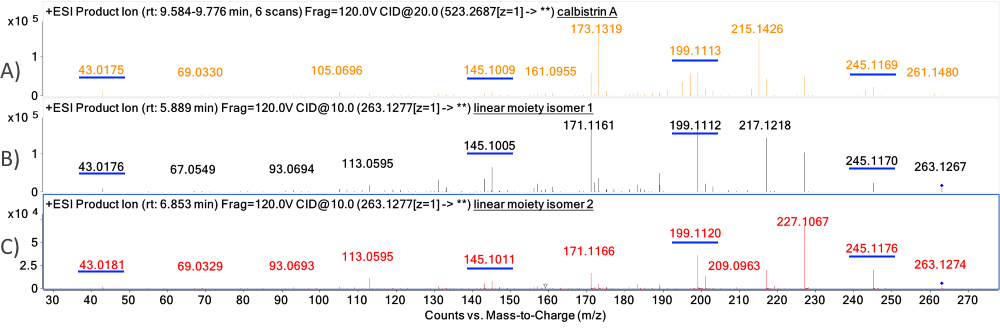 |
| --- |
| 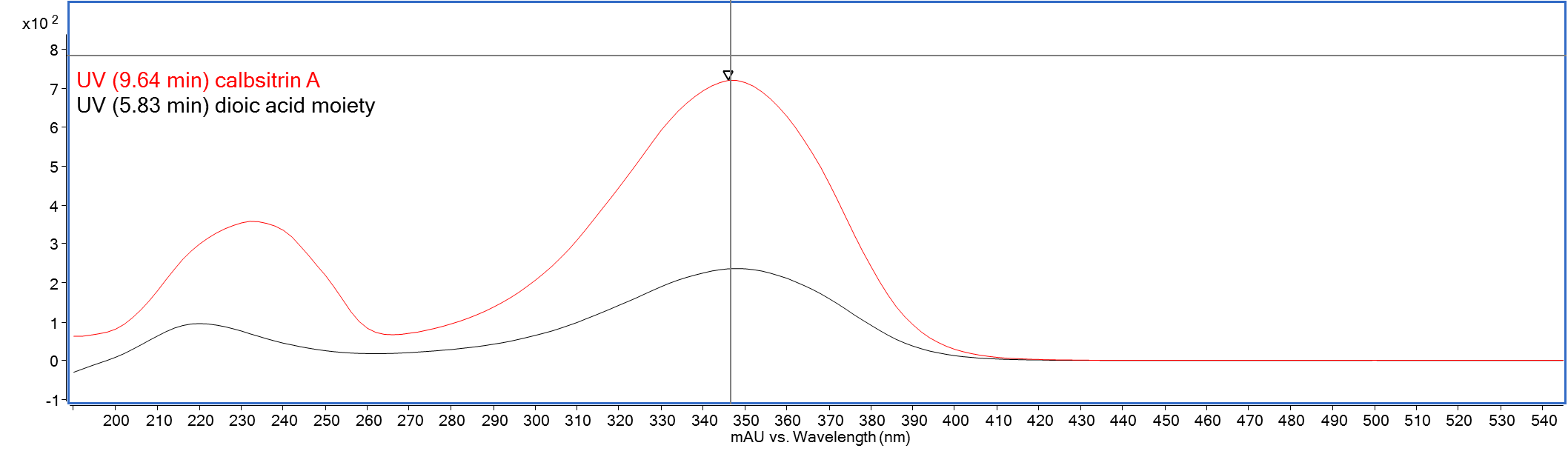 |

| **Transformation** | **Repetitions (new batch of protoplasts)** | **Obtained # colonies** | **Screened by colony PCR** | **Deletion or insertion observed** | **Purified for detection of calbistrin** |
| --- | --- | --- | --- | --- | --- |
| **Deletion of PKS pdec13/*calA*** | Yes (n=3) | 19, 0, 23 | 16, 0, 16 | 0, 0, 2 (12.5%) | 0, 0, 2 |
| **Deletion of TF *calC*** | No | 47 | 24 | 13 (54.1%) | 5 |
| **Deletion of MFS *calB*** | No | 183 | 38 | 5 (13.2%) | 2 |
| **Control A (3 µg pJAK-109)** | Yes (n=3) | 15, 0, 16 | 0 | n/a | n/a |
| **Control B(3 µg pCP-AMA-ergA)** | No | 30 | 0 | n/a | n/a |

Additional Information 5. Transformation experiments conducted during study. The overview contains the number of repetitions, the obtained number of transformants and colonies purified for analysis of calbistrin production.

| **BLASTP**  ***A. aculeatus*** | **Size (aa)** | **Conserved domain and notes** | **E- value** | **BLASTP**  ***P. decumbens* (best)** | **% identity** | **number of proteins with > 50% coverage** | **BLASTP**  ***A. versicolor* (best)** | **% identity** | **number of proteins with > 50% coverage** |
| --- | --- | --- | --- | --- | --- | --- | --- | --- | --- |
| XP_020058114.1 | 278 | pfam13649 - Methyltransferase | 1.17E-09 | OQD67221.1 | 30 | 1 | OJJ05901.1 | 58 | 7 |
| XP_020058115.1 | 347 | No putative conserved domains detected. | n/a | OQD74941.1 | 32 | 10 | OJJ07025.1 | 33 | 53 |
| XP_020058116.1 | 210 | DUF3328 super family | 3.14E-20 | No putative conserved Protein sequence detected | | | OJJ07031.1 | 32 | 1 |
| XP_020058117.1 | 245 | DUF3328 super family | 2.84E-33 | OQD76856.1 | 35 | 0 (18% for OQD76856.1) | OJJ05902.1 | 63 | 4 |
| XP_020058118.1 | 70 | No putative conserved domains detected. | n/a | No putative conserved Protein sequence detected | | | OJI98640.1 | 64 | 0 (20% for OJI98640.1) |
| XP_020058119.1 | 104 | No putative conserved domains detected. | n/a | No putative conserved Protein sequence detected | | | OJI95849.1 | 46 | 2 |
| XP_020058120.1 | 171 | cl25409, Short-chain dehydrogenase/reductase (SDR) | 3.19E-51 | OQD74315.1 | 94 | 8 | OJI95847.1 | 61 | 30 |

**Additional information 6.** Putative proteins of additional ORFs identified within the calbistrin cluster of *A. aculaetus* and BLASTP results against the *P. decumbens* and *A. versicolor* genomes.

Additional information 7. Gene synteny analysis *P. decumbens* and *Colletotrichum tofieldiae*. calA cluster (part of scaffold 13) from *P. decumbens* and selected part of scaffold 170 from *C. tofieldiae*. No further significant synteny was detected between other regions of the scaffolds.


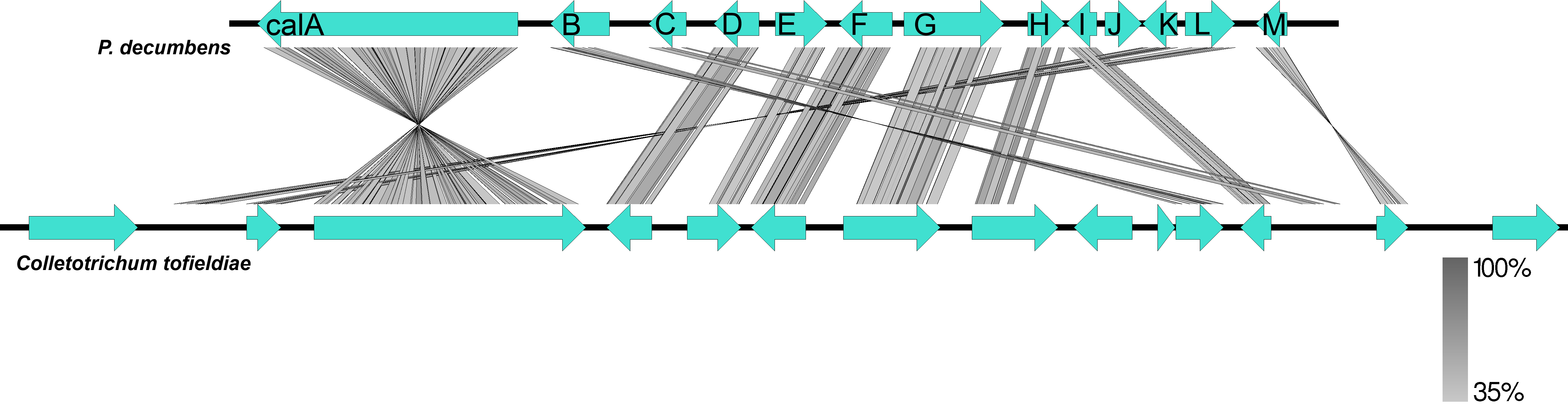


Additional information 8. Results of transcriptomics data analysis (RNA-seq) of P. decumbens grown in liquid CM, supporting calbistrin production and liquid DM, where calbistrin is not produced. Expression results were normalized for fragments per kilobase of exon per million reads mapped (FPKM). Differential expression analysis was computed for complex medium relative to defined medium using DESeq2.

| Gene | log2 FoldChange (log2FC) | adjusted p-value | FPKM CM_run 1 | FPKM CM_run 2 | FPKM CM_run 3 | FPKM DM_run 1 | FPKM DM_run 2 | FPKM DM_run 3 |
| --- | --- | --- | --- | --- | --- | --- | --- | --- |
| PENDEC_c013G04345 | -0.89286 | 1.84E-01 | 44 | 34 | 51 | 107 | 105 | 43 |
| PENDEC_c013G05431 | -0.62931 | 6.33E-01 | 64 | 54 | 62 | 29 | 41 | 246 |
| calM | 3.37505 | 1.43E-06 | 18479 | 14184 | 10747 | 694 | 1678 | 246 |
| calL | 3.73218 | 5.08E-09 | 3313 | 2766 | 1809 | 119 | 223 | 41 |
| calK | 3.09810 | 1.10E-04 | 4914 | 4116 | 2687 | 220 | 503 | 43 |
| calJ | 1.83873 | 9.18E-04 | 1331 | 1439 | 987 | 384 | 403 | 152 |
| calI | 2.98965 | 1.43E-05 | 5163 | 4222 | 3085 | 308 | 662 | 110 |
| calH | 3.77871 | 3.60E-08 | 7641 | 5887 | 3959 | 270 | 416 | 60 |
| calG | 2.72723 | 2.61E-03 | 3639 | 2903 | 2017 | 173 | 467 | 21 |
| calF | 3.02686 | 1.00E-04 | 1916 | 1541 | 1156 | 107 | 211 | 21 |
| calE | 3.39921 | 1.39E-05 | 6196 | 4787 | 3518 | 216 | 467 | 38 |
| calD | 3.29274 | 2.85E-05 | 8772 | 6374 | 4526 | 307 | 715 | 63 |
| calC | 1.01449 | 2.83E-01 | 194 | 270 | 183 | 89 | 167 | 25 |
| calB | 1.80813 | 9.78E-04 | 73 | 58 | 53 | 24 | 13 | 9 |
| calA | 3.17153 | 8.75E-08 | 1543 | 1334 | 1057 | 144 | 144 | 38 |
| PENDEC_c013G02827 | 0.11450 | 8.74E-01 | 184 | 222 | 161 | 212 | 137 | 174 |
| PENDEC_c013G03462 | 0.72867 | 2.81E-01 | 21 | 25 | 19 | 13 | 17 | 7 |
| PENDEC_c001G04327 (actin) | 0.06299 | 9.55E-01 | 3181 | 3119 | 2829 | 4422 | 2614 | 1675 |

Additional information 9. Sequence of constructed selection marker cassette for overexpression of *ergA* (Pc22g15550). The *PgpdA* promoter (blue) from A. nidulans was used to drive expression of the squalene epoxidase *ergA* (green) originating from *P. chrysogenum*. As terminator, the acetamidase terminator *TamdS* (from *A. nidulans*, red) was used. Underlined sequence: silent mutation to remove BsaI restriction site; Black sequence: backbone pICH57751.

| 5’….. **TGTGAAGACAAACTAGAATTCGAGCTCGGAGTGGATCCCCCGGGCTGCAGGAATTCGAGCTCTGTACAGTGACCGGTGACTCTTTCTGGCATGCGGAGAGACGGACGGACGCAGAGAGAAGGGCTGAGTAATAAGCCACTGGCCAGACAGCTCTGGCGGCTCTGAGGTGCAGTGGATGATTATTAATCCGGGACCGGCCGCCCCTCCGCCCCGAAGTGGAAAGGCTGGTGTGCCCCTCGTTGACCAAGAATCTATTGCATCATCGGAGAATATGGAGCTTCATCGAATCACCGGCAGTAAGCGAAGGAGAATGTGAAGCCAGGGGTGTATAGCCGTCGGCGAAATAGCATGCCATTAACCTAGGTACAGAAGTCCAATTGCTTCCGATCTGGTAAAAGATTCACGAGATAGTACCTTCTCCGAAGTAGGTAGAGCGAGTACCCGGCGCGTAAGCTCCCTAATTGGCCCATCCGGCATCTGTAGGGCGTCCAAATATCGTGCCTCTCCTGCTTTGCCCGGTGTATGAAACCGGAAAGGCCGCTCAGGAGCTGGCCAGCGGCGCAGACCGGGAACACAAGCTGGCAGTCGACCCATCCGGTGCTCTGCACTCGACCTGCTGAGGTCCCTCAGTCCCTGGTAGGCAGCTTTGCCCCGTCTGTCCGCCCGGTGTGTCGGCGGGGTTGACAAGGTCGTTGCGTCAGTCCAACATTTGTTGCCATATTTTCCTGCTCTCCCCACCAGCTGCTCTTTTCTTTTCTCTTTCTTTTCCCATCTTCAGTATATTCATCTTCCCATCCAAGAACCTTTATTTCCCCTAAGTAAGTACTTTGCTACATCCATACTCCATCCTTCCCATCCCTTATTCCTTTGAACCTTTCAGTTCGAGCTTTCCCACTTCATCGCAGCTTGACTAACAGCTACCCCGCTTGAGCAGACATCACAATGATGACCTTGCTCAATGGCCACGCCTCCCTCTCCCCGGCCGCTCAACGCCGCATCGAACACCATGAAGCTGATATTGTAATTGTGGGTGCCGGAGTCCTCGGCTGTGCACTTGCAGTTGCACTGGGAAATCAAGGGCGCAGTGTAATCCTGCTGGAGAAGTCGTTGGAAGAGCCCAACCGTATTGTTGGTGAACTACTCCAACCGGGAGGTGTTCAGGCGCTCGAGCAACTAGGGCTACGGGATTGTCTGGAAGATATCGATGGCATCAATGTGAAGGGATACTGGGTTACTTACTTTGGGGAGCCGGTTCTCCTCGAATACCCCAAGTCCAGCCCAACCTCGCCTACACCGTTGGGACGAGCCTTCCACCATGGCCGATTCGTGATGAAACTACGGGCAGCCGCTCTGTCGTGCCCCAATGTCACGGTGGTCGAAACCAAGGTTACCGGCCTCATCACATCCTCATATACTCAGGAAGTCCTTGGAGTCGAGTGTATAACCAAGGATGTCAAGGACTGCTACTTCGGTCAACTCACCGTGGCCGCCGACGGTTATAACTCCGTTTTCCGCAAGGAACACCACCAGTATACACCCAAACGGCGCTCAAAGTTCCATGGCTTGGAGCTCATCGATGCTAAACTGCCCGCTCCCAACACTGGTCATGTGCTTCTCAGCGATAATCCACCCGTACTTATGTACCAGATCGGCACCCATGAAACCCGAATCCTTATCGATATCCCTGATAACCTGCCTGCGGCCTCGGTCAAGAACGGTGGTGTCAAGGGTTACATGCGAAACAACATCCTGCCCAAGCTCCCAGAGGGTGTCCAGCAGTCGTTCTCCGATGCCCTAGAGAAGGGCCAGCTACGGTCAATGCCGAACTCGTTCCTGCCTGCCTCGGCCAACAAGACACCCGGCTTGATGATTCTGGGCGATGCGCTTAACATGCGGCACCCTCTCACAGGTGGAGGCATGACTGTGGCGCTCAACGATGTTTGTGTCATCCGCGAATTGCTCAGCCCAGAGCGCGTGCCCAACCTCTCCAATACAGGCCTTGTTCTCGAGCAGCTCGCAGAGTTCCACTGGAAACGAAAGAACTCATCTTCGGTCATCAATATCCTCGCACAGGCTCTCTATGCGCTCTTCGCTGCAGACAGTACGCACGCTCACTCTTCAGGTTAATTTGCAATAACTAACTGTTCATTCAACAGACTATTACCTCAAAGCTCTTCAGCGTGGCTGTTTCCGCTACTTCCAGATTGGACCAGTTGGCGGCCCTGTCGGGTTGTTAGCCGGTCTAATCAAGAAACCCCTCGTCCTTGTCAGCCACTTCTTCTCCGTTGCCTTCCTCGCCATCTGGGTCCACATATGCGATACCCCTCTGTCCAAGCTATTCCTAGCCCCATACTACGCCGTCATGATATTGTACACAGCCTCTGTCGTGATTTTGCCATACATCTGGACCGAGATCTGGTACTGACTAATAAGTGTCAGATAGCAATTTGCACAAGAAATCAATACCAGCAACTGTAAATAAGCGCTGAAGTGACCATGCCATGCTACGAAAGAGCAGAAAAAAACCTGCCGTAGAACCGAAGAGATATGACACGCTTCCATCTCTCAAAGGAAGAATCCCTTCAGGGTTGCGTTTCCAGTCTAGACACGTATAACGGCACAAGTGTCTCTCACCAAATGGGTTATATCTCAAATGTGATCTAAGGATGGAAAGCCCAGAATATTGGCTGGGTTGATGGCTGCTTCGAGTGCAGTCTCATGCTGCCACAGGTGACTCTGGATGGCCCCATACCACTCAACCCATGGTACGAGCGGTACGCTTTACTTGTCTTCTGCACGAAGTGGTTT….3’** |
| --- |

| **Name** | **protospacer (5’-n_20_-3’)** | **Quality score** |
| --- | --- | --- |
| sgRNA_Pdec13_1 | GTGAACAGCAACAAGACTCG | 1.38 |
| sgRNA_Pdec13_2 | GGGTCCCGAGAACTACATTA | 0.20 |
| sgRNA_Pdec13_3 | GAAGAACTGCGAATCGAAGG | 1.23 |
| sgRNA_Pdec13_4 | GCGATCTGGTGAATCTCGCG | 1.31 |
| sgRNA_calC _1 | GGACCCGGGTCGGACTGCTG | 1.04 |
| sgRNA_calC _2 | GTAGATGGGACGAGTGGAGG | 0.77 |
| sgRNA_calC _3 | GGAGGCCAGCGCAAGCCAGG | 1.80 |
| sgRNA_calC _4 | GTGGATGGATGACAGCGGGC | 1.19 |
| sgRNA_calB_1 | GGACCTGGAGGAAGAGTAGG | 1.44 |
| sgRNA_calB_2 | GTTAGTAAGAAAAAAGCTCG | 1.41 |
| sgRNA_calB_3 | GGGCGATGTTGAGAGTCCGG | 1.31 |
| sgRNA_calB_4 | GCTGGTGAAACAAAGCTAGC | 1.02 |

Additional Information 10. List of protospacers used in this study. The score for protospacer quality was calculated using sgRNA-scorer 2.0.

Additional Information 11. List of primers used in this study.

| **Name** | **Sequence (5’ to 3’)** | **Purpose** |
| --- | --- | --- |
| ergA-BsaI-FW_1 | GGAAGACAAAATGATGACCTTGCTCAATGGCCACG | Cloning of *P. chrysogenum* *ergA* via MoClo technique |
| ergA_1_R | GGAAGACAATTCATGGGTGCCGATCTGGTAC |  |
| ergA_2_FW | GGAAGACAATGAAACCCGAATCCTTATCGATATCCCTG |  |
| ergA_2_R | GgaagacAAaagcTCAGTACCAGATCTCGGTCCAGATG |  |
| cPCR_calA_FW | TGGAGAGACACCCTTACACTTACCC | Diagnostic (colony) PCR *calA* (PKS) |
| cPCR_calA_R | AGCCTACGTAGAGGGGCTCAGC |  |
| cPCR_calC_FW | ACGAGGAATATACGTCCATATCAAAGG | Diagnostic (colony) PCR *calC* (TF) |
| cPCR_calC_R | TTGATGTTGATGTTCTATCTCCGATCG |  |
| cPCR_calB_FW | TAAGGCTCAGCCATCTTTACTGAGG | Diagnostic (colony) PCR *calB* (MFS) |
| cPCR_calB_R | CAGTAGGAAAGTAACATCATTTGCAGACC |  |
| qPCR_calA_F | CGTAGTTCAGGTAGTTGCTGTGC | qPCR expression of calA |
| qPCR_calA_R | CTTTACACCAGCAACTCTGAGTGC |  |
| qPCR_calB_F | AGAATTCCAGGCAGTGTATTCACTCC | qPCR expression of calB |
| qPCR_calB_R | GTGCTGCTGGCTCTCTACTACG |  |
| qPCR_calC_F | TTCGCCAGTGAGTTATATTTTGAATCC | qPCR expression of calC |
| qPCR_calC_R | GACTCCTACATTCCTTAATACCCTGG |  |
| qPCR_calF_F | CTGACGATGACCTTTTTCTGAGTCG | qPCR expression of calF |
| qPCR_calF_R | CCTGATCAGAATGGCACCCAGG |  |
| qPCR_actin_F | CGCCTTCTACGTCTCCATTCAGG | qPCR expression of ɣ-actin |
| qPCR_actin_R | CGGTGGTGGAGAAAGTGTAGCC |  |

Additional Information 12. Workflow for construction and screening after loss-of-function mutants in *P. decumbens*. Initial screening was based on survival on selection plates (phleomycin or terbinafine) followed by diagnostic colony PCR reactions (cPCR-xxx_FW/R primers). If PCR products indicated a shift in band size, clones were further analyzed by sequencing.


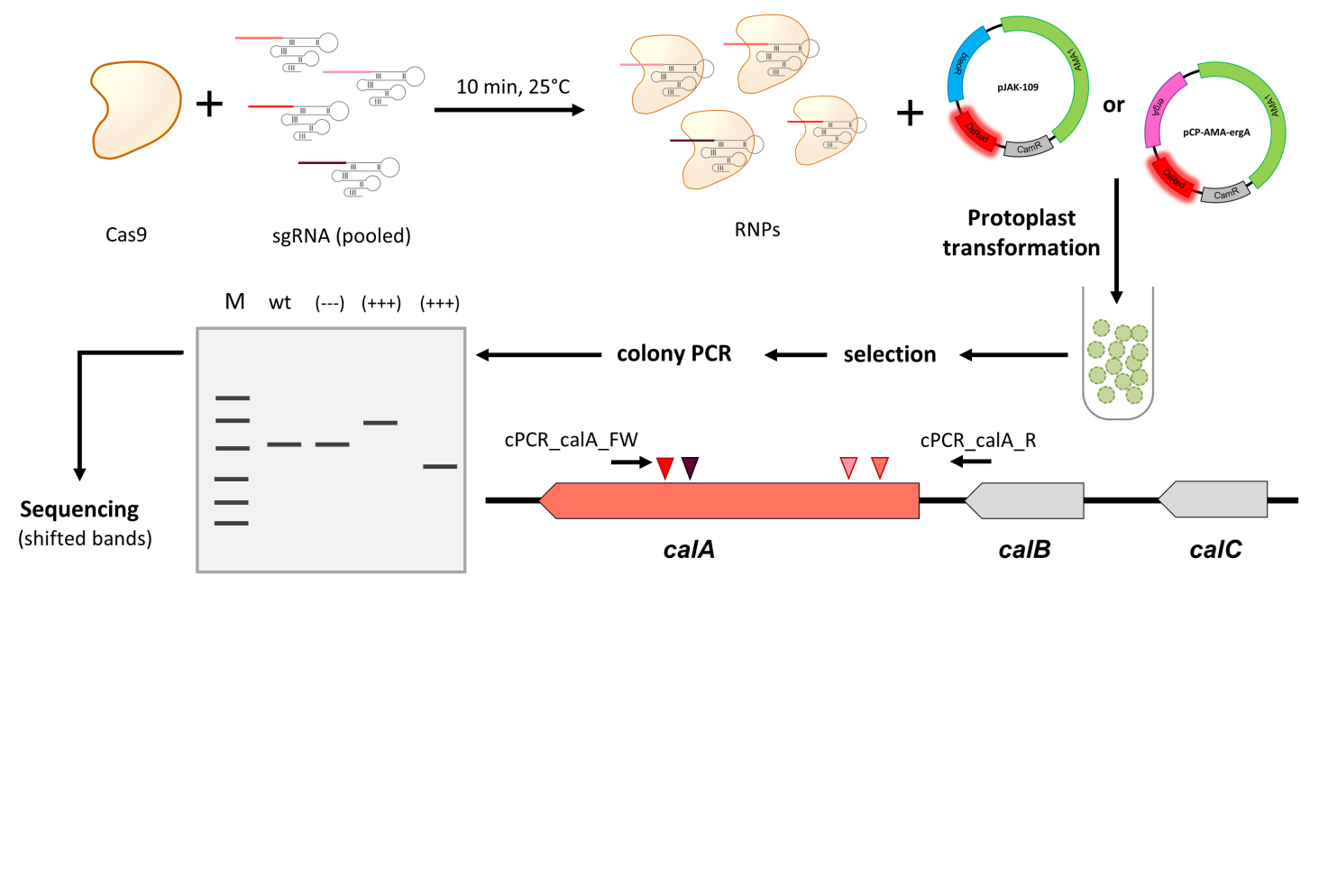


Additional Information 13. Results of PCR based screening for disruption of *calA* in *P. decumbens*. A) Schematic representation of targeting sites of sgRNAs and primers used for colony PCR. B) Results of colony PCRs. Two of fourteen transformants displaying a different amplicon size compared to the wild type strain (1206 bp) and these were analyzed further. C) Summary of obtained mutants, occured mutation and sgRNAs that cutted. D) Position of sgRNAs and sequencing traces in parental *P. decumbens* strain. Red line indicates the *calA* gene. Blocks highlight sgRNA including PAM. E) Sequencing results of ∆*calA*-1 and ∆*calA*-2. Both colonies show a partial loss of the protospacer motif, framing a removed sequence of 414 bp.


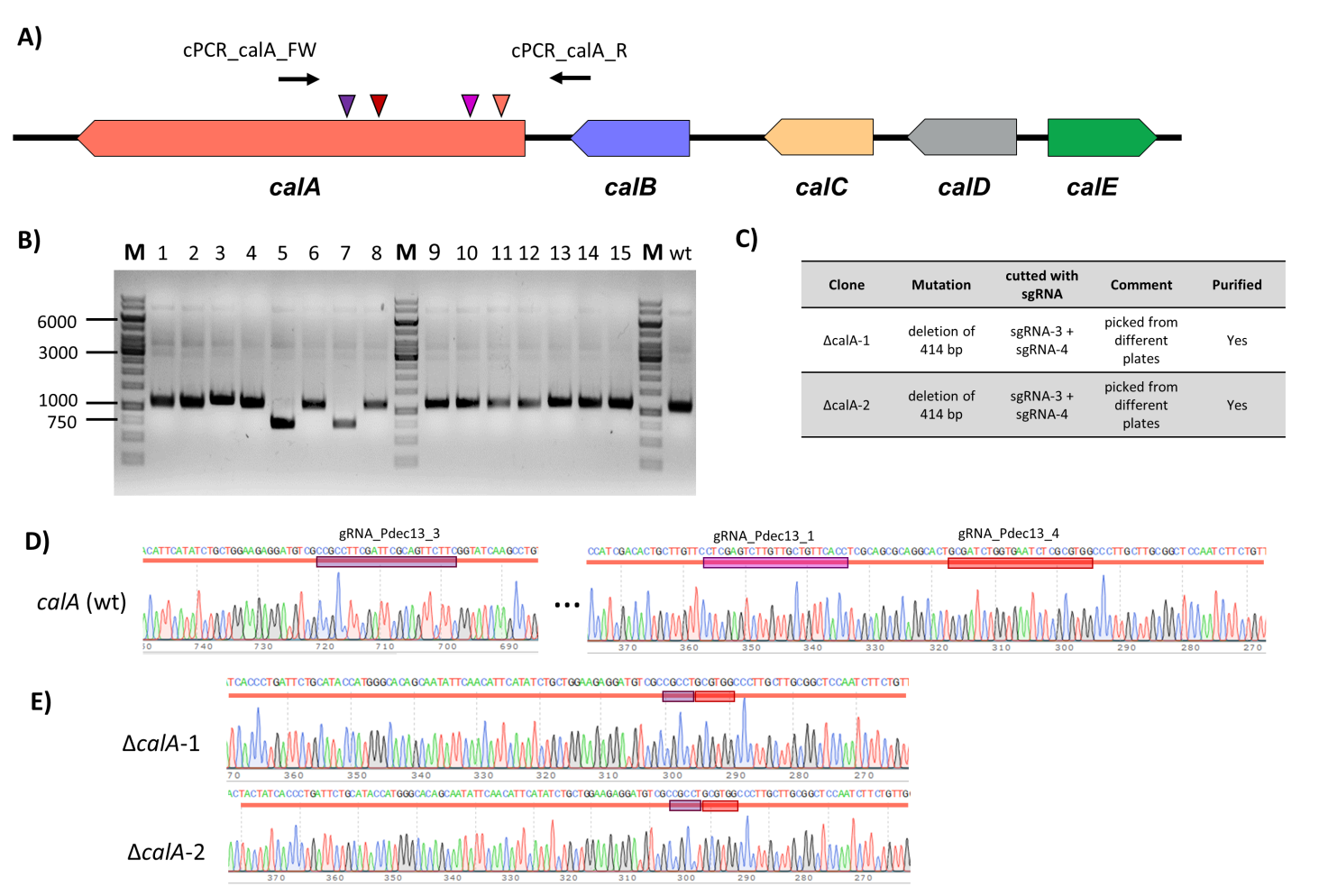


Additional Information 14. Results of PCR based screening for disruption of *calB* in *P. decumbens*. A) Schematic representation of targeting sites of sgRNAs and primers used for colony PCR. B1) and B2) Results of diagnostic colony PCR reactions. Four out of nineteen transformants displaying a different band size compared to the parental strain (2285 bp) and these were analyzed further. Asteriks indicating a faint band. C) Summary of obtained mutants, occured mutation and sgRNAs that cutted. D) Position of sgRNAs and sequencing traces in parental *P. decumbens* strain. Blue line indicates the *calB* gene. Blocks highlight sgRNA including PAM. E) Sequencing results of ∆*calB*-11 and ∆*calB*-24. Both colonies show a partial loss of the protospacer motif after cutting occurred. Pink line in ∆*calB*-24 represents the integrated Terbinafine cassette located on pCP-AMA-ergA.


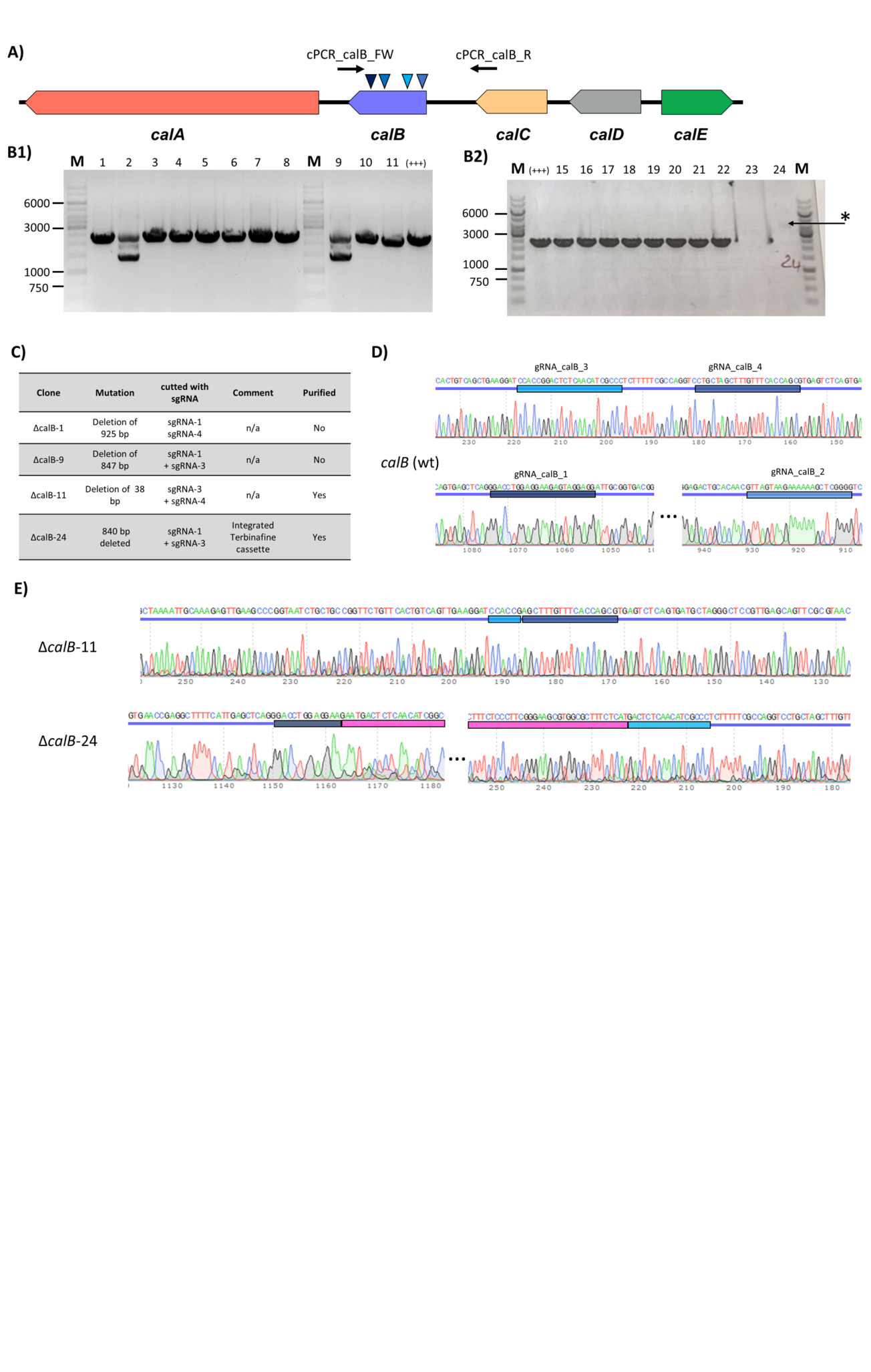


Additional Information 15. Results of PCR based screening for disruption of *calC* in *P. decumbens*. A) Schematic representation of targeting sites of sgRNAs and primers used for colony PCR. B) Results of diagnostic colony PCRs screening: Five transformants displaying a different band size compared to the parental strain (1485 bp) were analyzed further C) Summary of analyzed mutants, occured mutation and sgRNAs that cutted. D) Position of sgRNAs and sequencing traces in parental *P. decumbens* strain. Yellow line indicates the *calB* gene. Blocks highlight sgRNA including PAM. E) Sequencing results of ∆*calC*-2 and ∆*calC*-7. Both colonies show a partial loss of the protospacer motif after cutting occurred. Pink line in ∆*calC*-7 represents the integrated Terbinafine cassette located on pCP-AMA-ergA.


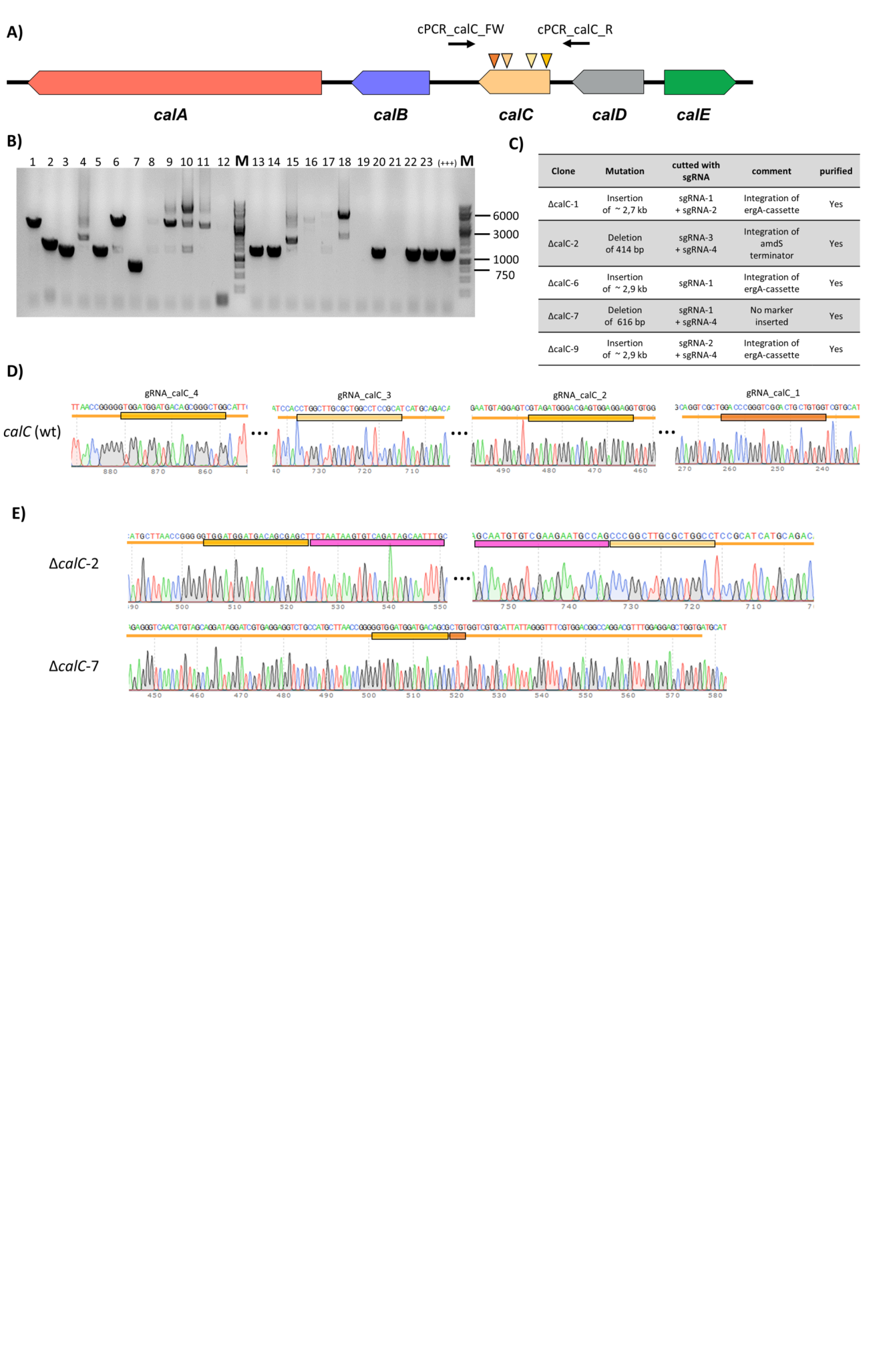


**Additional Information 16: Amino acid sequence for Softberry FGENESH predicted gene models for *calG, calJ* and *calM****.*

**>calG**

MATEKTPDWKIVDLQWAPDLGPVPVQDAMRPTRESQGEQEMIARMWVMCAIQMQDKLCAAKCTKQHFERYRSWLTAEYERFKQPGYPQVPDSRELVDMSNDARLAAMNKLREGVKNTYMWPVIEGPWRVYDNVVDIVEGRVKLVKVLLQDGLLEKFYDWANGLSEVRPLFNRMGRSNSSLRILEIGAGTGGTTARALEGLKSDDGELLYSSYEFTDISPLFFDAARRRFEGYSNIEYRALDISRNAVEQGFEAGAYDLVIASNVLHATPCLVDTLKNVRLLLKPNGFLFNQELSPPGKYVDFMVGLLPGWWLGDADGRAEGPCIPPEEWHRRLEQAGFEGLHAVGFDSDPPYYYNANMIARPAVNA

**>calJ**

MSDFDALLANYTSKETPKVHGVICKCVDRHGICPTPHLAKRDLRLTLLGNEIYSKVAGYDSVLPGASPLREDVVLKVASATKLITSIALLQCIDKGLIDLDEPVTKVLPEFDQKQILTDVSGSDLVLEPSKTPITARHLLTHTSGLGYPFTHRLLRLRAEVRNRAGVSPSLRVTERYEMPLVFEPGTGWLYGCSLDWAGVIVSRLHGGISLEQYFVENIWQRLGLSEPFPCFNIARHPEYNARVMGGAIQTPEGRLQPKDHWAFDNPEDQDGGSGLSCTTKDYVAVLADLVSDSPKLLKPATIAEMFTPQLEAKSPGVQMLLGLRPAWDTVSGPIAENAINHGLGGVLCMDDVPEIDQPKGMLGWGGASNIVWWVNRELRVAGFFATQQAPFGNPSVTRLVNAWKKDFWAQFKTIDHA

**>calM**

MNPVNTKPYQLSADATWFVTGCSTGIGRAIASHVASQPGHRLIATARDPSSLSYLDDDNPAILKLAMDVTNPSSVNAAFKAAADYFGDKYYIDVVVNNAGYSLSGDTESVTEHEMHDEFETNFFGTVRVTLKAIEVMRQSKDHRGGLIFNISSLAGICAFPGHAFYHASKFAVEGWSESVAREMHPDWNIHFCIVEPSAVKTNFETTSKKRTQPHEAYAGADMPARQLETFVKKGLEAGVGFEPSAVANVLYKVASRNEKVPLRLPLSATAVKLITAKLQVQLQDLETVSELSAIDVHQVQFKV
